# Clathrin-nanoparticles deliver BDNF to hippocampus and enhance neurogenesis, synaptogenesis and cognition in HIV/neuroAIDS mouse model

**DOI:** 10.1038/s42003-022-03177-3

**Published:** 2022-03-17

**Authors:** Gordana D. Vitaliano, Jae K. Kim, Marc J. Kaufman, Christopher W. Adam, Gonzalo Zeballos, Abinaya Shanmugavadivu, Sivan Subburaju, Jay P. McLaughlin, Scott E. Lukas, Franco Vitaliano

**Affiliations:** 1 Department of Psychiatry, Harvard Medical School, Boston, MA 02115 USA; 2 Brain Imaging Nanotechnology Group, McLean Hospital, Belmont, MA 02478 USA; 3 McLean Imaging Center, McLean Hospital, Belmont, MA 02478 USA; 4 Department of Pharmacodynamics, University of Florida, Gainesville, FL 32610 USA; 5 ExQor Technologies Inc, Boston, MA 02114 USA

**Keywords:** Regeneration and repair in the nervous system, Regenerative medicine

## Abstract

Brain derived neurotrophic factor (BDNF) promotes the growth, differentiation, maintenance and survival of neurons. These attributes make BDNF a potentially powerful therapeutic agent. However, its charge, instability in blood, and poor blood brain barrier (BBB) penetrability have impeded its development. Here, we show that engineered clathrin triskelia (CT) conjugated to BDNF (BDNF-CT) and delivered intranasally increased hippocampal BDNF concentrations 400-fold above that achieved previously with intranasal BDNF alone. We also show that BDNF-CT targeted Tropomyosin receptor kinase B (TrkB) and increased TrkB expression and downstream signaling in iTat mouse brains. Mice were induced to conditionally express neurotoxic HIV Transactivator-of-Transcription (Tat) protein that decreases BDNF. Down-regulation of BDNF is correlated with increased severity of HIV/neuroAIDS. BDNF-CT enhanced neurorestorative effects in the hippocampus including newborn cell proliferation and survival, granule cell neurogenesis, synaptogenesis and increased dendritic integrity. BDNF-CT exerted cognitive-enhancing effects by reducing Tat-induced learning and memory deficits. These results show that CT bionanoparticles efficiently deliver BDNF to the brain, making them potentially powerful tools in regenerative medicine.

## Abstract (web summary)

Vitaliano et al. show in mice that engineered clathrin triskelia (CT) conjugated to BDNF (BDNF-CT) and delivered intranasally increases hippocampal BDNF concentrations 400-fold above that achieved previously with intranasal BDNF alone. They also show that it enhance neurogenesis, synaptogenesis and cognition in a mouse model of HIV/neuroAIDS, highlighting its potential in regenerative medicine.

## Introduction

To advance diagnosis and treatment of central nervous system (CNS) disorders, it is crucial to develop technologies that deliver adequate concentrations of biologics to the CNS. The intranasal (i.n.) route of drug administration exploits direct transport pathways to the brain, and is a safe and noninvasive drug delivery method that bypasses the blood brain barrier (BBB), avoids first-pass metabolism, and minimizes systemic exposure and peripheral side effects^1,2^. Drugs administered i.n. can enter the CNS via paracellular or intracellular routes from the nasal mucosa, and along the trigeminal and olfactory nerve tracts. However, large, hydrophilic or charged proteins cannot readily pass the nasal barrier and enter into the brain^3^. To overcome this barrier, clathrin nanoparticles (NPs) capable of transporting large molecules into the CNS were bioengineered^4^. Clathrin is a natural protein found in bacteria, fungi, plants, animals, and humans^5,6^. Clathrin-mediated endocytosis provides a natural pathway for internalizing extracellular molecules and nutrients^5,6^ and transporting molecules across the BBB^7^. Clathrin also plays a role in trafficking of synaptic vesicles^8^, cell signaling^9^, mitosis^10^, and proliferation^10^. Clathrin triskelion, a mono-unit composed of three heavy and three light chains, can self-assemble into fullerene-shaped structures called clathrin cages (CCs), which are ~100-fold stiffer than liposomes^11^. CCs are resistant to trypsin digestion^12^. Furthermore, CT or CCs can serve as stable nanoplatforms onto which multiple molecules, such as therapeutic drugs or imaging agents, can be attached or encapsulated through chemical modifications^4^. These properties make clathrin an ideal nanoparticle for brain delivery of diagnostic or therapeutic biologics, such as neurotrophic factors (NTFs).

NTF biologics have been studied as treatments for neurodegenerative disorders (e.g., Alzheimer’s disease, Parkinson’s and Huntington’s disease), neuroinflammatory diseases (e.g., amyotrophic lateral sclerosis and multiple sclerosis), HIV-associated neurocognitive disorder (HAND), stroke, traumatic brain injury, and psychiatric disorders (e.g., depression)^13^. One of the most studied NTFs is brain-derived neurotrophic factor (BDNF)^14^. BDNF is widely distributed in the CNS and plays a central role in cell metabolism, survival, growth, and maintenance^15–17^. Its ability to modulate synaptic plasticity^18–20^ and learning and memory processes^21–24^, as well as its protective effects in adult brain^25^, have been extensively documented. Accordingly, early intervention with BDNF treatment may help prevent or impede the development of neurodegenerative disorders^26^. However, several properties of BDNF, including its charge, extremely short plasma half-life on the order of minutes^27^, poor pharmacokinetic (PK) profile, and poor BBB penetrability^28^, limit its use as a therapeutic. Accordingly, parenteral^28^, or i.n.^29^ BDNF administration results in minimal CNS delivery.

Different strategies have been developed either to deliver BDNF into the brain or to increase its CNS concentrations including: 1) direct CNS infusions^30–32^, 2) cell and gene therapy via injection of stem cells^33,34^ or viral vectors^25,35,36^, 3) polymer implants that release BDNF^37^, 4) dual drug loaded lipid NPs^38^, and 5) ultrasound enhanced BDNF delivery^39^. However, these methods require invasive procedures that are not suitable for routine clinical practice. Noninvasive BDNF delivery methods using NPs capable of crossing the nasal barrier or the BBB have achieved varying degrees of success (for review, see ref. ^40^). For example, BDNF was transported across the BBB via anti-transferrin receptor antibodies^41^ or poloxamor-188 polymeric NPs^42^. Also, BDNF encapsulated in polymer^43^ or fused with cell-penetrating peptides and packaged in adenovirus-associated virus NPs^44^ passed the nasal barrier. However, modifications that increase NP stability and/or CNS penetrability often increase immunogenicity or toxicity^40^. By contrast, clathrin is a natural, stable, nonimmunogenic, biocompatible, and biodegradable protein that does not require enhancements to penetrate CNS after i.n. or i.v. delivery^4^. Therefore, clathrin is an excellent candidate as a nanocarrier for BDNF delivery to the brain.

In the present study, we tested whether a novel clathrin NP efficiently delivers BDNF into the brain after i.n. administration, and whether BDNF-CT NP exerts beneficial molecular, cellular, and behavioral effects in an iTat mouse model of HIV/neuroAIDS^45,46^. Downregulation of BDNF has been linked to HIV-associated neurodegeneration in animals and humans^47,48^. In HIV patients, decreased BDNF levels in cerebrospinal fluid (CSF) are strongly correlated with increased severity of neurological disease^49,50^. Based on severity of disease, HAND includes: asymptomatic neurocognitive impairment (ANI), mild neurocognitive disorder (MND), and HIV associated dementia (HAD). HIV Transactivator-of-Transcription (Tat) protein is a HIV regulatory protein that promotes viral replication. Tat is released by HIV-infected cells, interacts with uninfected cells (e.g., neurons) and is linked to HAND^51^ and neurodegeneration^52^. Tat protein has been found in post-mortem brain tissues of HIV patients^53^ and in the CSF of patients treated with antiretroviral therapy (ART)^54^. In animal models, Tat expression induces inflammation, apoptosis, gliosis^55^, and oxidative stress^56^. Tat expression also reduces gray matter density^57^, and shortens neurite outgrowth by down-regulation of BDNF^58^. Conditional Tat protein expression also induces learning and memory deficits in iTat mice^59–61^. BDNF reverses Tat-induced neurotoxicity in vitro^62^, but BDNF’s effects in vivo have not been tested in iTat mice. We hypothesized that BDNF-CT would target CNS TrkB receptors, activate downstream signaling pathways, increase hippocampal newborn cell survival and proliferation, enhance hippocampal neurogenesis, synaptogenesis and dendritic integrity, and ameliorate learning and memory deficits. The hippocampal brain region was selected for this study because human postmortem studies showed the highest viral concentrations^63,64^ and a maximal degree of microglial activation and neuroinflammation in the temporal lobe of individuals with HIV^65^. Increased plasma viral load and lower CD4+ T-cell counts were consistently associated with smaller hippocampal volumes in HIV-positive adults worldwide, and therefore it is important to protect and restore hippocampal structure and function early in the course of HIV-1 infection^66^.

## Results

### BDNF-CT characterization

**Fig. 1:**
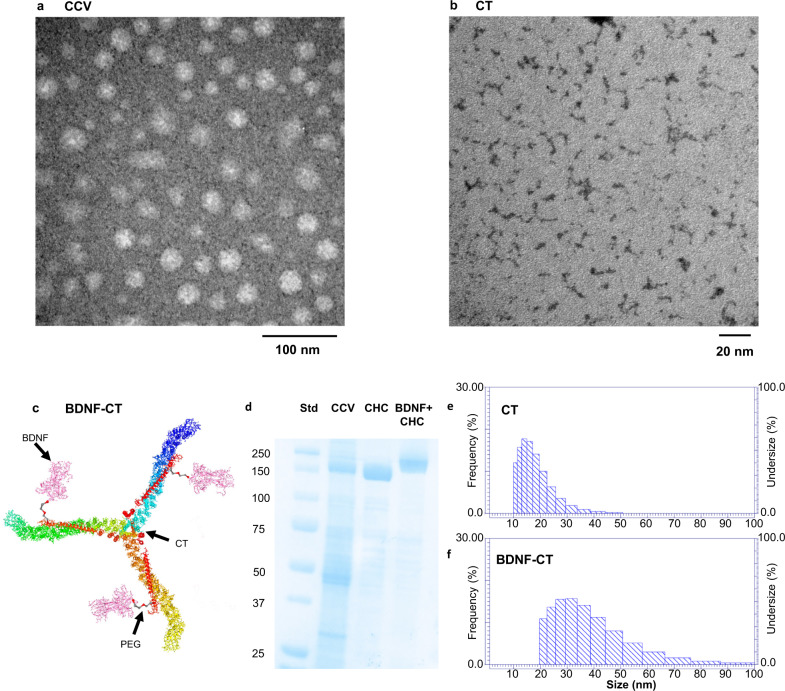
BDNF-Clathrin (BDNF-CT) nanoparticle purification and characterization. Transmission electron microscopy (TEM) images show (**a**) clathrin coated vesicles (CCVs) isolated from pig brains, and (**b**) purified clathrin triskelia (CT) with attached metal (Gd) negatively stained with 1% uranyl acetate. (**c**) A graphical representation of a clathrin triskelion with three heavy chains (CHCs, yellow, blue and green) and three light chains (CLCs, red). One BDNF molecule is conjugated to the each CHC via PEG and NP solution had 2.25 mg/ml of protein (2 mg/ml of CT with 0.25 mg/ml of BDNF). (**d**) SDS-PAGE shows CCV, CT, and BDNF-CT protein bands. The CHC molecular weight increased by 30 kDa following BDNF conjugation to CT. (**e**) Dynamic light scattering (DLS) studies show the mean hydrodynamic radius (*R*_H_) of unconjugated CT to be 16.8 ± 5.6 nm. (**f**) The mean *R*_H_ increased to 35.1 ± 12.6 nm after BDNF PEGylation and subsequent conjugation to CT.

Transmission electron microscopy (TEM) images showed clathrin coated vesicles (CCVs) isolated from pig brains (Fig. 1a), and clathrin triskelia (CT) isolated from CCVs (Fig. 1b).

A diagram of three recombinant human BDNF molecules conjugated to CT via Polyethylene glycols (PEGs) was created using PyMOL 2.5 and is shown in Fig. 1c. The PBS solution of BDNF-CT had 2.25 mg/mL of protein (2 mg/mL of CT with 0.25 mg/mL of BDNF). Molar ratio of BDNF to CT was 3:1. SDS-PAGE analysis revealed a 30 kDa molecular weight increase of the clathrin heavy chain (CHC) that confirmed successful conjugation of BDNF to clathrin triskelion at a 3:1 (BDNF:CT) molar ratio (Fig. 1d). Dynamic light scattering (DLS) studies revealed that the mean CT hydrodynamic radius (*R*_H_) was 16.8 ± 5.6 nm, consistent with the previously reported value of 16.9 nm^67^ (Fig. 1e). A single triskelion has three legs that are flexible, puckered, bent, stretched, close together, or extended. Electron microscopy has shown that triskelion legs can vary from 35 to 62 nm in total length after straightening^68,69^. Atomic force microscopy also confirmed that the legs are flexible along their entire length^70^. Therefore, there is variability in the measurements of triskelion size. BDNF conjugation to CT increased the *R*_H_ to 35.1 ± 12.6 nm (Fig. 1f). These results indicate that BDNF-PEGs formed a stable complex with CT, and that free molecules of BDNF-PEGs were not present in the NP solution.

### BDNF-CT targeted TrkB receptors

**Fig. 2:**
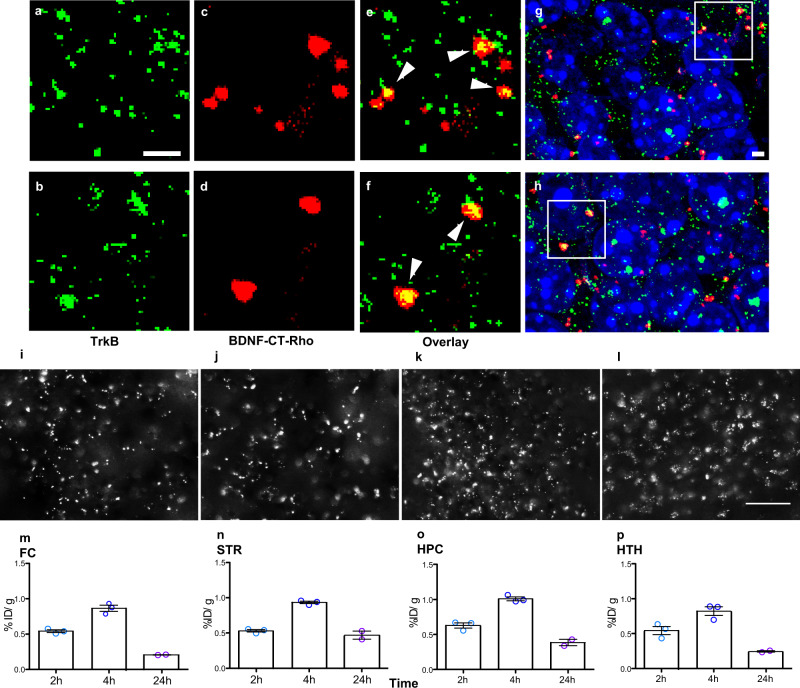
CT efficiently delivered BDNF to TrkB receptors in the mouse brain. Tat+ mice were given a single dose of i.n. BDNF-CT-Rho (0.3 mg/kg of BDNF and 2.4 mg/kg of CT). Brains were collected after 4 h. Brain sections were probed for TrkB and visualized using Alexa Fluor 488. Confocal microscopy shows TrkB receptors (green) (**a**, **b**) and intranasally delivered BDNF-CT-Rho (red) nanoparticles (**c**, **d**). BDNF-CT-Rho are co-localized with TrkB receptors (yellow) (**e**, **f**). Images were taken from two DG locations (box; **g**, **h**). Scale bars are 2 µm. Representative images of the BDNF-CT-Rho are shown in the frontal cortex (FC) (**i**), striatum (STR) (**j**), hippocampus (HPC) (**k**), and hypothalamus (HTH) (**l**). Brains were also collected from Tat+ mice at 2, 4, and 24 h after i.n. ^[3]^H-BDNF-CT NP administration. Tissue concentrations of radioactive ^[3]^H-BDNF-CT were assessed. The peak concentrations of BDNF-CT in the FC (**m**), ST (**n**), HPC (**o**), and HTH (**p**) were achieved 4 h after administration. Data are represented as the mean % of the injected dose per gram (% ID/g) of tissue (*n* = 3/group and for 24 h *n* = 2/group). Error bars represent S.E.M. The scale bar is 50 µm.

Tat+ mice were given a single dose of i.n. BDNF-CT-Rhodamine. Brains were collected after 4 h. Immunostaining with anti-TrkB antibody revealed a robust TrkB immunoreactivity (IR) (green) in the hippocampus (Fig. 2a, b). Fluorescent rhodamine (Rho) signals (red) were observed abundantly throughout the hippocampus in animals that had received BDNF-CT-Rho (Fig. 2c, d). The merged confocal image revealed regions positive for both TrkB and BDNF-CT-Rho throughout the hippocampus. Clear overlapping of the two labels (yellow) was observed at higher magnifications (Fig. 2e, f), and at multiple regions within the dentate gyrus (DG) (Fig. 2g, h), indicating that intact BDNF-CT targeted TrkB receptors in vivo.

### BDNF-CT brain distribution

Qualitative assessment of mouse brains 4 h after i.n. delivery of rhodamine labeled BDNF-CT showed punctate fluorescent deposits in multiple brain regions (Fig. 2i–l). Quantitative assessment of [^3^H]-BDNF-CT concentrations in brain regions at different time points indicated that peak [^3^H]-BDNF-CT concentrations were achieved 4 h after i.n. administration (Fig. 2m–p). For example, peak [^3^H]-BDNF-CT concentrations were found in the frontal cortex (FC) (0.864 ± 0.074% injected dose per gram (% ID/g) of tissue), striatum (STR) (0.932 ± 0.029% ID/g), hippocampus (HPC) (1.008 ± 0.048% ID/g), and hypothalamus (HTH) (0.821 ± 0.1087% ID/g).

### CT are required for BDNF delivery to TrkB receptors and downstream signaling

#### Assessments of mBDNF and proBDNF levels

**Fig. 3:**
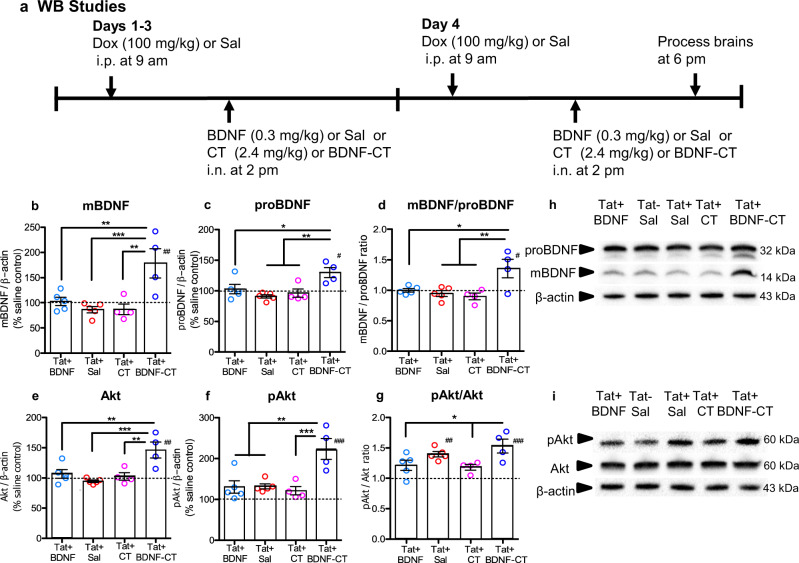
CT are required for BDNF delivery to the hippocampus and increased Akt expression and signaling. Tat+ mice received i.p. Dox and one of four i.n. treatments (BDNF, Sal, CT, or BDNF-CT) daily for 4 days (**a**). Global control (Tat−) mice received i.p. and i.n. saline (Tat-/Sal). The hippocampal tissue shows increased mBDNF (**b**), proBDNF (**c**), mBDNF/proBDNF ratio (**d**), Akt (**e**), pAkt (**f**), and the pAkt/Akt ratio (**g**) in Tat+ mice that received BDNF-CT vs. other treatments (*P < 0.05, **P < 0.01 and ***P < 0.001), as well as vs. saline treated Tat- mice (^#^*P* < 0.05 and ^##^*P* < 0.01, ^###^*P* < 0.001)^.^ Tat+ mice treated with BDNF-CT or Sal had higher pAkt/Akt ratios (**g**) compared to Tat- controls but levels of Akt and pAkt were significantly higher in BDNF-CT vs. Sal treated Tat+ mice indicating enhanced Akt expression and signaling only with BDNF-CT NPs. Representative WB images are shown (**h**, **i**). Data are shown as the mean % change from Tat- control (Tat-/Sal) represented by dotted lines (*n* = 4–5 per group, five cohorts were processed in parallel). Error bars represent S.E.M.

BDNF-CT treatment (Fig. 3a) significantly increased mature BDNF (mBDNF) levels compared to controls (*F*_(4,18)_ = 8.069, *P* = 0.0007, *n* = 4–5/group; Fig. 3b). Post hoc analysis indicated that hippocampal mBDNF levels were higher in induced iTat (Tat+) mice that had received BDNF-CT compared to saline (*P* < 0.001), CT alone (*P* < 0.01), and unconjugated BDNF (*P* < 0.01). As expected, neither unconjugated BDNF nor CT alone increased mBDNF levels (*P* > 0.05; Fig. 3b). In Tat+ mice, the normalized mean hippocampal mBDNF levels (expressed as percent differences from saline-treated Tat− controls, Fig. 3b) were as follows: 178.2 ± 58% with BDNF-CT; 86.95 ± 21.4 % with CT; 86.19 ± 13.7% with saline and 102.6 ± 18.2% with native BDNF treatments. BDNF-CT also increased proBDNF levels (*F*_(4,18)_ = 6.239, *P* = 0.0025, *n* = 4–5/group; Fig. 3c). Post hoc analysis revealed increased hippocampal proBDNF levels in Tat+ mice that had received BDNF-CT compared to saline (*P* < 0.01), CT alone (*P* < 0.01), and unconjugated BDNF (*P* < 0.05). Again, proBDNF levels were not altered either by unconjugated BDNF or by CT (*P* > 0.05; Fig. 3c).

BDNF-CT increased the mBDNF to proBDNF ratio (*F*_(4,18)_ = 6.554, *P* = 0.002; Fig. 3d). Post hoc analysis revealed an increased mBDNF to proBDNF ratio in Tat+ mice that had received BDNF-CT compared to saline (*P* < 0.01), CT alone (*P* < 0.01) and unconjugated BDNF (*P* < 0.05).

Levels of mBDNF (*P* < 0.01), proBDNF (*P* < 0.05), and the mBDNF to proBDNF ratio (*P* < 0.05) were higher in Tat+ mice that had received BDNF-CT compared to saline-treated iTat (Tat−/Sal) control mice.

#### Assessment of BDNF-CT induced Akt expression and signaling

BDNF-CT increased hippocampal Akt levels (*F*_(4,18)_ = 8.874, *P* = 0.0004, *n* = 4–5/group; Fig. 3e). Akt levels were higher in Tat+ animals that had received BDNF-CT compared to saline (*P* < 0.001), CT alone (*P* < 0.01) and unconjugated BDNF (*P* < 0.01) (Fig. 3e). Similarly, BDNF-CT delivery increased hippocampal phosphorylated protein kinase B (pAkt) levels (*F*_(4, 18)_ = 11.72, *P* < 0.0001, *n* = 4–5/group; Fig. 3f). pAkt levels were higher in Tat+ mice treated with BDNF-CT compared to saline (*P* < 0.01), CT alone (*P* < 0.001) and unconjugated BDNF (*P* < 0.01) (Fig. 3f).

Neither CT nor unconjugated BDNF increased Akt or pAkt levels compared to saline (*P* > 0.05).

Also, levels of Akt (*P* < 0.01) and pAkt (*P* < 0.001) were higher in Tat+ mice that had received BDNF-CT compared to saline-treated Tat− control mice (Fig. 3e, f).

BDNF-CT increased the pAkt to Akt ratio (*F*_(4,18)_ = 9.314, *P* = 0.0003; Fig. 3g). Post hoc analysis revealed an increased pAkt to Akt ratio in Tat+ mice that had received BDNF-CT compared to CT alone (*P* < 0.05) and unconjugated BDNF (*P* < 0.05). Tat+ mice treated with BDNF-CT (*P* < 0.001) or saline (*P* < 0.01) had higher pAkt to Akt ratios compared to saline-treated Tat− control mice (Fig. 3g) indicating increased activation of Akt signaling pathway. However, levels of both Akt and pAkt were significantly higher in BDNF-CT vs. Sal treated Tat+ mice indicating enhanced Akt expression and signaling only with BDNF-CT treatment. Representative WB images for mBDNF, proBDNF, Akt, and pAkt are shown in the Fig. 3h, i.

#### Assessment of BDNF-CT induced TrkB expression and signaling

The full-length TrkB protein expression was measured by Western blot (WB) analysis of 145 kDa bands. BDNF-CT increased hippocampal TrkB levels (*F*_(2,9)_ = 47.98, *P* < 0.0001, *n* = 4/group; Supplementary Fig. 1a). Full-length TrkB levels were higher in Tat+ animals that had received BDNF-CT compared to saline treated Tat+ (*P* < 0.001) and Tat− (*P* < 0.001) mice. Similarly, BDNF-CT delivery increased hippocampal phosphorylated TrkB (pTrkB) levels (*F*_(2,9)_ = 255.3, *P* < 0.0001, *n* = 4/group; Supplementary Fig. 1b). Full-length pTrkB levels were higher in Tat+ mice treated with BDNF-CT compared to saline treated Tat+ (*P* < 0.001) and Tat− (*P* < 0.001) mice.

BDNF-CT increased the pTrkB to TrkB ratio (*F*_(2,9)_ = 39.78, *P* < 0.0001, *n* = 4/group; Supplementary Fig. 1c). Post hoc analysis revealed an increased pTrkB to TrkB ratio in Tat+ mice that had received BDNF-CT compared to saline treated Tat+ (*P* < 0.001) and Tat− (*P* < 0.001) mice (Supplementary Fig. 1c). Representative WB images for pTrkB and TrkB are shown in the Supplementary Fig. 1d.

#### Assessment of Tat expression

Hippocampal Tat protein expression was measured by WB analysis of 22 kDa bands^71^. Daily Dox treatment (100 mg/kg/d, i.p.) over 4 days increased Tat expression in Tat+ mice (*F*_(2,9)_ = 28.20, *P* = 0.0001, *n* = 4/group; Supplementary Fig. 2a). In the hippocampus, Tat levels were lower in Tat− controls compared to saline-treated Tat+ mice (*P* < 0.001) and BDNF-CT treated Tat+ mice (*P* < 0.001). Hippocampal Tat protein expression was not significantly different between Tat+ mice that had received BDNF-CT compared to saline (*P* > 0.05, Supplementary Fig. 2a). A faint 22 kDa band was observed in Tat- mice given saline, which may indicate a low level of Tat expression (Supplementary Fig. 2b).

### BDNF-CT enhanced newborn cell survival, proliferation, and neurogenesis in the granule cell layer (GCL) of dentate gyrus (DG)

#### Assessment of newborn cell survival

**Fig. 4:**
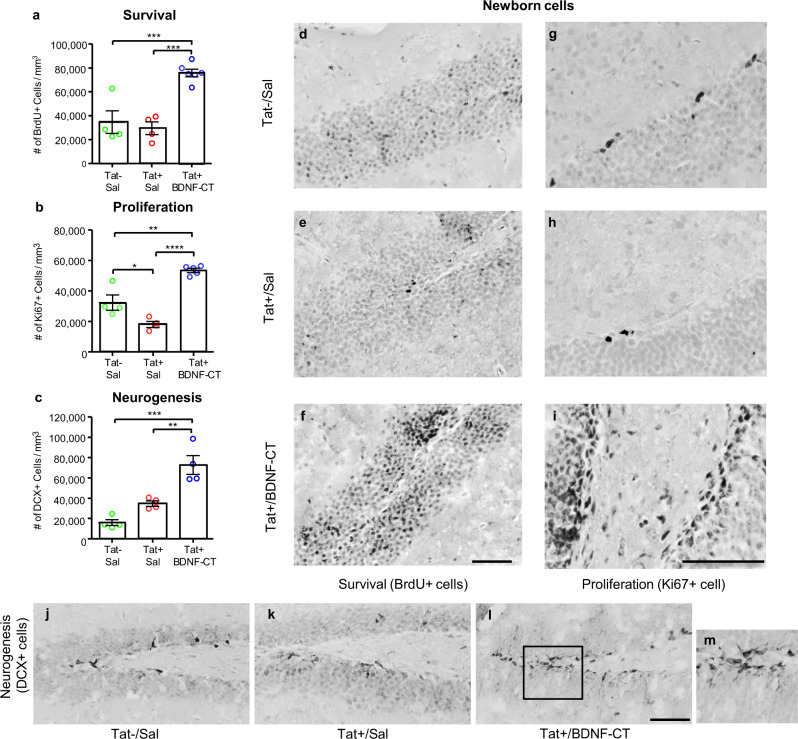
BDNF-CT increased newborn cell survival, proliferation, and neurogenesis. Tat+ mice received i.p. Dox (100 mg/kg) and i.n. saline or BDNF-CT (0.3 mg/kg of BDNF and 2.4 mg/kg of CT) daily for 7 days. Tat− controls received i.p. and i.n. saline. All mice received BrdU (50 mg/kg, i.p. every 12 h) on days 1 and 2 and were sacrificed on the 7th day. Hippocampal sections containing the granule cell layer (GLC) were evaluated for BrdU+, Ki67+, and DCX+ cells using IHC with nickel enhanced DAB staining (black). Densities of BrdU+ cells (**a**), Ki67+ cells (**b**), and DCX+ cells (**c**) are higher in BDNF-CT-treated Tat+ mice vs. matched controls (Tat+ and Tat−) that received saline. Data are represented as the mean density. Error bars represent S.E.M. (*n* = 4–6 per group, *P < 0.05, **P < 0.01, ***P < 0.001, and ****P < 0.0001). Images represent BrdU+ (**d**–**f**), Ki67+ (**g**–**i**) or DCX+ (**j**–**l**) staining (black) in the hippocampal GCL following administration of Sal/Sal to Tat− mice, and Dox/Sal or Dox/BDNF-CT to Tat+ mice. Higher magnification of the marked area (**l**) with DCX+ cells are shown (**m**). Color saturation is 0% and scale bars are 50 µm.

BDNF-CT treatment enhanced the survival of newborn cells (*F*_(2,11)_ = 21.43, *P* = 0.0002, *n* = 4–6/group, Fig. 4a). In the GCL of DG, bromodeoxyuridine positive (BrdU+) cell densities were higher in Tat+ mice that had received BDNF-CT compared to saline treated Tat+ (*P* < 0.001) and Tat− mice (*P* < 0.001; Fig. 4a).

#### Assessment of newborn cell proliferation

BDNF-CT treatment increased the proliferation of newborn cells (*F*_(2,10)_ = 38.54, *P* < 0.0001, *n* = 4–5/group; Fig. 4b). In the GCL, Ki67-positive (Ki67+) cell densities were higher in Tat+ mice that had received BDNF-CT compared to saline treated Tat+ (*P* < 0.0001) and Tat− mice (*P* < 0.01). Notably, after 7 days of Tat induction, a decrease in Ki67 cell densities (*P* < 0.05) was observed in Tat+ versus Tat− mice.

#### Assessment of neurogenesis

BDNF-CT treatment increased neurogenesis (*F*_(2,9)_ = 24.69, *P* = 0.0002, *n* = 4/group; Fig. 4c). In the GCL, doublecortin-positive (DCX+) cell densities were higher in Tat+ mice that had received BDNF-CT compared to saline-treated Tat+ (*P* < 0.01) and Tat− mice (*P* < 0.001; Fig. 4c).

Representative images show BrdU+ (Fig. 4d–f), Ki67+ (Fig. 4g–i), and DCX+ (Fig. 4j–m) staining in the hippocampus.

#### Assessment of newborn cell differentiation into young neurons

**Fig. 5:**
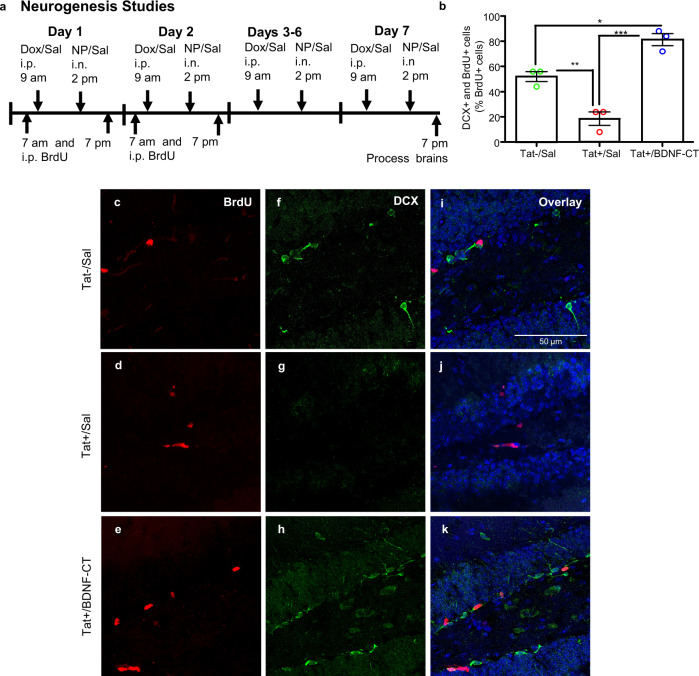
BDNF-CT rescues neurogenesis from Tat-induced toxicity in the hippocampus. Tat+ mice received i.p. Dox and i.n. saline or BDNF-CT nanoparticles (0.3 mg/kg of BDNF and 2.4 mg/kg of CT) daily for 7 days. Tat− controls received i.p. and i.n. saline. All mice received BrdU (50 mg/kg, i.p. every 12 h) on days 1 and 2 and were sacrificed on the 7th day (**a**). Hippocampal sections containing the granule cell layer were evaluated and double labeled cells (BrdU+ and DCX+) are expressed as the percentage of BrdU+ cells (**b**). The mean percentage was higher in BDNF-CT-treated Tat+ mice vs. matched controls (Tat+ and Tat−) that received saline, and was lower in Tat+ vs. Tat− mice. BrdU+ cells (red, **c**–**e**), DCX+ cells (green, **f**–**h**) and double labeled cells (BrdU+ and DCX+) (**i**–**k**) are shown. Nuclei (blue) were labeled with DAPI. Data are represented as the mean percentage. Error bars represent S.E.M. (*n* = 3 per group; *P < 0.05, **P < 0.01, and ***P < 0.001). Scale bars are 50 µm.

BDNF-CT treatment (Fig. 5a) enhanced differentiation of newborn cells into young neurons (*F*_(2,6)_ = 43.66, *P* = 0.0003, *n* = 3/group; Fig. 5b). In the GCL, BrdU+ cells (Fig. 5c–e), DCX+ cells (Fig. 5f–h) and double labeled cells (BrdU+ and DCX+) (Fig. 5i–k) were evaluated. The mean percentage of double-labeled cells (BrdU+ and DCX+) was significantly higher in Tat+ mice that had received BDNF-CT compared to saline-treated Tat+ (*P* < 0.001) and Tat− mice (*P* < 0.05). After 7 days of Tat induction, a decrease in the mean percentage of double-labeled cells (BrdU+ and DCX+) (*P* < 0.01) was found in Tat+ versus Tat− mice (Fig. 5b).

### BDNF-CT enhanced synaptogenesis and dendritic integrity in the hippocampal regions

**Fig. 6:**
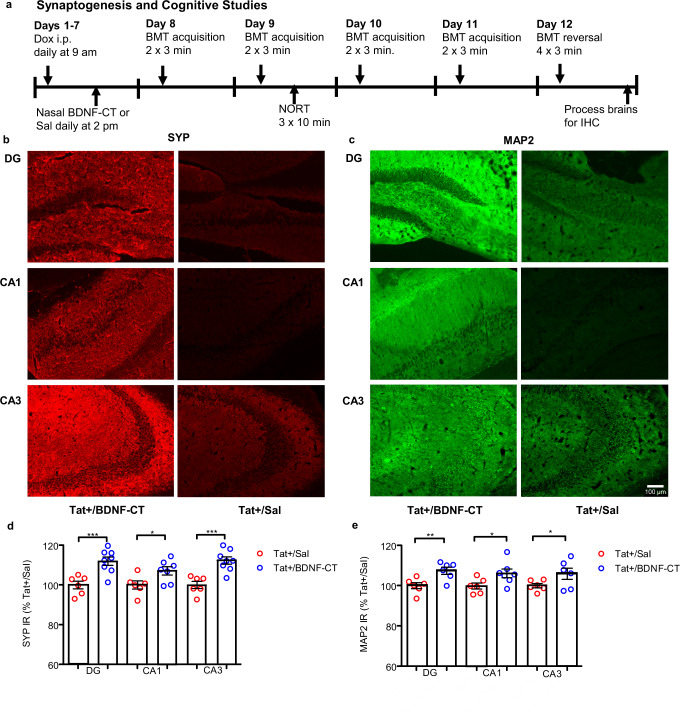
BDNF-CT enhanced synaptogenesis and dendritic integrity in the Tat+ mouse hippocampus. Tat+ mice received i.p. Dox (100 mg/kg) and i.n. saline or BDNF-CT (0.23 mg/kg of BDNF and 1.85 mg/kg of CT) daily for 7 days. Cognitive testing started on the 8th day and lasted for 5 days (**a**). Mice were sacrificed on day 12 and hippocampal dentate gyrus (DG), CA1 and CA3 sections were evaluated for SYP+ (**b**) (red) and MAP2+ (**c**) (green) staining. The SYP (**d**) and MAP2 (**e**) immunoreactivities (IR) increased in the hippocampal DG, CA1, and CA3 regions in BDNF-CT-treated Tat+ mice vs. saline treated controls. Data are shown as the mean % change from saline treated controls. Error bars represent S.E.M. (*n* = 6–8 per group; *P < 0.05, **P < 0.01, ***P = 0.001). Scale bars are 100 µm.

BDNF-CT administration (Fig. 6a) increased synaptogenesis (Fig. 6b) and dendritic integrity (Fig. 6c) in the hippocampus. The hippocampal synaptophysin (SYP) IR was higher in the DG (*t*-test, *n* = 6–8/per group, *P* = 0.0007), CA1 (*P* = 0.0177), and CA3 (*P* = 0.0003) regions of Tat+ mice that had received BDNF-CT compared to saline treated Tat+ controls (Fig. 6d). The hippocampal microtubule associated protein 2 (MAP2) IR also was increased in the DG (*t*-Test, *N* = 6–8/per group, *P* = 0.0052), CA1 (*P* = 0.0195) and CA3 (*P* = 0.0396) regions of Tat+ mice that had received BDNF-CT compared to saline-treated Tat+ controls (Fig. 6e).

### BDNF-CT enhanced learning, memory, and cognitive flexibility

#### Recognition memory

**Fig. 7:**
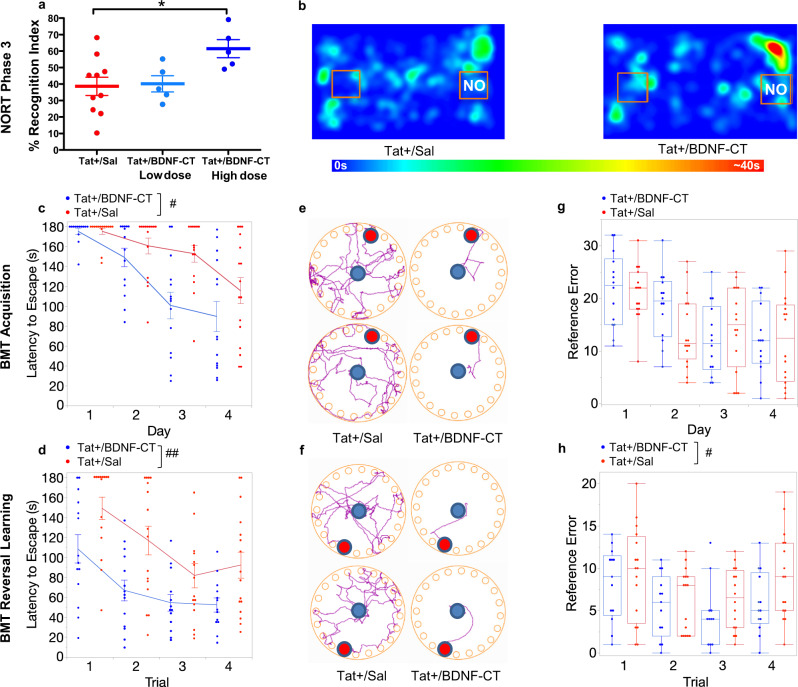
BDNF-CT ameliorated Tat-induced learning and memory deficits and enhanced cognitive flexibility in the Novel object recognition test (NORT) and Barnes maze test (BMT). Tat+ mice received i.p. Dox (100 mg/kg) and either i.n. saline or BDNF-CT daily for 7 days. Testing procedures started on the 8th day and lasted for 5 days. (**a**) NORT Phase 3 Recognition Index (RI) was significantly higher in Tat+ mice treated daily with a high dose (0.3 mg/kg of BDNF and 2.4 mg/kg of CT) but not a low dose (0.15 mg/kg of BDNF and 1.2 mg/kg of CT) of NPs compared to saline (*n* = 5–10 per group). (**b**) Representative heat maps show time spent in different locations of the NORT testing chamber. In the BMT, the latency to locate and enter the escape hole in the acquisition (**c**) and reversal learning (**d**) phases was lower in Tat+ mice treated with BDNF-CT vs. saline. Representative diagrams show paths from the starting position (blue dot) to the escape hole (red dot) on day 3 of acquisition phase (**e**) and trials 2 and 4 of reversal learning phase (**f**) (top and bottom, respectively). The number of reference errors (**g**, **h**) was significantly lower in BDNF-CT compared to saline treated Tat+ mice only in the reversal learning phase (**h**) of BMT. Data are represented as the mean. Error bars represent S.E.M. Overall treatment differences in the BMT are indicated by ^#^*P* < 0.05 and ^##^*P* < 0.01. Between-subjects post hoc analysis in the NORT is indicated by *P < 0.05.

There was an effect of treatment (*F*_(2,17)_ = 4.138, *P* = 0.0343, *n* = 5–10/group; Fig. 7a) on recognition index (RI) of the Novel Object Recognition Test (NORT). Tat+ mice that had received BDNF-CT spent more time exploring the novel object. Post hoc analysis revealed dose-related effects. The % RI values were higher in animals that were given a high dose (0.3 mg/kg of BDNF and 2.4 mg/kg of CT; *P* < 0.05), but not a low dose of BDNF-CT (0.15 mg/kg of BDNF and 1.2 mg/kg of CT; *P* > 0.05), when compared to saline treated Tat+ controls. Representative heat maps show time spent in different locations of the NORT testing chamber (Fig. 7b).

#### Spatial learning and memory

During the acquisition phase (*N* = 30; Fig. 7c) of the Barnes Maze Test (BMT), there was an overall effect of treatment (*F*_(1,27.7)_ = 4.336, *P* = 0.0467) and day (*F*_(3,25)_ = 16.186, *P* < 0.0001) on mean escape latency. Tat+ mice (*n* = 14) given BDNF-CT spent less time locating and entering the escape chamber than saline-treated Tat+ controls (*n* = 16) (Fig. 7e). There was an overall effect of day (*F*_(3,83.1)_ = 11.788, *P* < 0.0001) but not treatment on numbers of errors (Fig. 7g). However, there was an overall effect of treatment (*F*_(1,27.2)_ = 7.243, *P* = 0.012) on average speed, which increased in BDNF-CT vs. saline treated Tat+ mice (Supplementary Fig. 3a). Overall effects of day (*F*_(3,82.2)_ = 22.664, *P* < 0.0001) were found for path efficiencies (Supplementary Fig. 3b).

#### Cognitive flexibility

In the BMT reversal learning phase (*N* = 30; Fig. 7d), there were overall effects of treatment (*F*_(1,30.9)_ = 11.285, *P* = 0.0021) and trial (*F*_(3,76.9)_ = 11.5, *P* < 0.0001) on escape latency. Tat+ mice (*n* = 14) given BDNF-CT performed better than saline treated Tat+ controls (*n* = 16) (Fig. 7f). We also found an overall effect of treatment (*F*_(1,107)_ = 5.112, *P* = 0.0258) and trial (*F*_(3,107)_ = 3.693, *P* = 0.0142) on numbers of reference errors (Fig. 7h). Overall effects of trial (*F*_(3,24.5)_ = 5.459, *P* = 0.0051) were found for average speed (Supplementary Fig. 3c). We also found overall effects of treatment (*F*_(1,43.7)_ = 4.881, *P* = 0.0325) and trial (*F*_(3,75.8)_ = 3.053, *P* = 0.0335) on path efficiency (Supplementary Fig. 3d).

#### Correlations between cognitive performance and markers of synaptogenesis and dendritic integrity

**Fig. 8:**
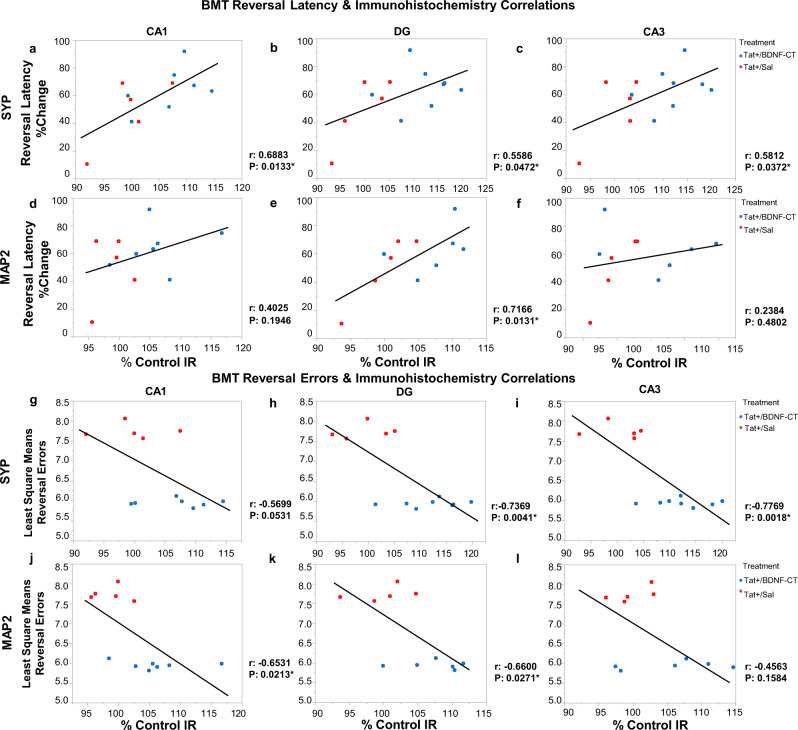
Improvements in memory and cognitive flexibility during BMT reversal leaning task are associated with enhanced hippocampal synaptogenesis and dendritic integrity. The mean latency percent change from the maximum time allowed to find an escape hole in the BMT reversal learning phase (reversal latency %Change) versus the mean CA1, DG, and CA3 hippocampal SYP (**a**–**c**) and MAP2 (**d**–**f**) immunoreactivities (IR, normalized to Tat+/Sal controls) are shown. The reference errors least square means versus the mean CA1, DG, and CA3 hippocampal SYP (**g**–**i**) and MAP2 (**j**–**l**) immunoreactivities (normalized to Tat+/Sal controls) are shown. Correlation Coefficients (*r*) were determined by calculating Pearson product-moment correlations for CA1, DG, and CA3 hippocampal regions. Significant correlations (*P* < 0.05) are indicated by *.

Correlations between BMT variables (e.g., the mean latency percent change from the maximum time allowed to find an escape hole and reference errors least square mean (LSM)) and hippocampal markers for synaptic density (SYP) and dendritic integrity (MAP2) were assessed in Tat+ mice treated with BDNF-CT or saline (Fig. 8).

Significant correlations were found between improved reversal learning task performance (% change in the latency to escape) and increased SYP IR in the CA1 (*r* = 0.6883, *P* = 0.0133), DG (*r* = 0.5586, *P* = 0.0472) and CA3 (*r* = 0.5812, *P* = 0.0372) regions of the hippocampus (Fig. 8a–c). Similar relationships existed between improved reversal learning task performance and MAP2 IR in the DG region (*r* = 0.7166, *P* = 0.0131) (Fig. 8e).

During the reversal learning task, inverse relationships were found between performance errors LSM (Fig. 8) and SYP IR in the DG (*r* = −0.7369, *P* = 0.0041) and CA3 (*r* = −0.7769, *P* = 0.0018) regions of the hippocampus. Also, there was a tendency for lower numbers of performance errors LSM to correlate with enhanced SYP IR in the CA1 region (*r* = −0.5699, *P* = 0.0531) (Fig. 8g). In addition, significant correlations were found between reversal learning task performance errors LSM and MAP2 IR in the CA1 (*r* = −0.6531, *P* = 0.0213) and DG (*r* = −0.6600, *P* = 0.0271) regions of the hippocampus (Fig. 8j, k).

### BDNF-CT did not exhibit toxic effects on motor functions

BDNF-CT treatment did not exhibit significant effects on average speed (Supplementary Fig. 4a) and total distance traveled (Supplementary Fig. 4b) in the open field (OF) test. The average speed and distance traveled were not significantly different (*P* > 0.05) in BDNF-CT vs. saline treated healthy C57BL/6J mice (Supplementary Fig. 4).

## Discussion

Our findings demonstrate that CT nanoparticles efficiently transported BDNF to hippocampus after i.n. administration and that BDNF concentrations were sufficient to induce beneficial molecular, cellular and behavioral effects in mice. BDNF-CT targeted TrkB receptors, increased newborn cell survival/proliferation and neurogenesis in the granule cell layer of dentate gyrus, and enhanced synaptogenesis and dendritic integrity. BDNF-CT attenuated learning and memory deficits induced by conditional Tat protein expression in Tat+ mice, most likely, as a direct consequence of the molecular effects noted above.

Intranasal BDNF-CT, but not BDNF or CT alone, increased hippocampal mBDNF levels, indicating that BDNF conjugation to CT is necessary for efficient i.n. delivery of BDNF to the brain. Intranasal BDNF-CT administration also increased proBDNF expression. The proBDNF also may have contributed to the higher mBDNF levels since mBDNF is formed by extracellular plasmin cleavage of proBDNF^72^, and because mBDNF stimulates its own production by increasing proBDNF synthesis and cleavage via a TrkB mediated mechanism^73^. The mBDNF/proBDNF ratio was higher in BDNF-CT treated Tat+ mice compared to saline-treated Tat+ and Tat− controls. The high mBDNF/proBDNF ratio may be functionally significant since mBDNF exerts pro-survival effects and enhances memory formation and storage^23^. ProBDNF binds to pan-neurotrophin receptor p75 (p75^NTR^) with high affinity and elicits biological effects opposing those of mBDNF^74^. While mBDNF induces long-term potentiation (LTP)^75–78^ and neurogenesis^79^, proBDNF prompts long-term depression^80^, and neuronal apoptosis^81^. Further, conversion of proBDNF into mBDNF regulates memory formation^82^ and is impaired in patients with HIV-associated dementia^48^. Therefore, the mBDNF/proBDNF ratio plays an important role in cell survival and cognitive functioning^82,83^.

BDNF binding to TrkB receptors elicits receptor dimerization and auto-phosphorylation, which activates intracellular pathways including the phosphatidyl inositol-3 kinase (PI3K)/protein kinase B (Akt) pathway^84^. BDNF not only induces Akt phosphorylation^85^, but also upregulates Akt^86^. The selective increases in pAkt and Akt levels by i.n. BDNF-CT treatment versus other treatments in our study confirms delivery of sufficient concentrations of intact BDNF for PI3K/Akt pathway activation. Tat+ mice treated with BDNF-CT or saline had higher pAkt to Akt ratios compared to saline-treated Tat- mice indicating PI3K/Akt pathway activation. HIV Tat can increase Akt signaling via multiple mechanisms^87^. However, levels of both Akt and pAkt were significantly higher in BDNF-CT vs. Sal treated Tat+ mice, indicating that only BDNF-CT treatment enhanced Akt expression and signaling. By increasing Akt levels, BDNF-CT kept the pAkt/Akt ratio in the normal range, which is important for maintaining normal cell functions and preventing Akt pathway dysregulation^88^.

Our results also show that biological activity of BDNF is preserved even after PEGylation and crosslinking to CT. The results from our confocal microscopy studies indicate that there was abundant and punctate BDNF-CT-Rho staining in DG that co-localized with TrkB staining. Brain distribution of BDNF-CT-Rho corresponds to the previously reported distribution of BDNF in the CNS^89^. These results further confirm delivery of NPs and selective targeting of TrkB receptors. Moreover, BDNF-CT increased full-length TrkB expression and signaling. The full length TrkB receptor was assessed because it contains an intracellular tyrosine kinase domain that rapidly transmits the effects of BDNF binding to a downstream network. Our results confirmed that BDNF-CT NP binds to full-length TrkB and triggers its downstream signaling in vivo.

We elected to use the noninvasive i.n. route for BDNF-CT administration because it circumvents the BBB and provides a direct pathway into the brain parenchyma^1,2^. The i.n. delivery efficiency of native BDNF to the brain is very low because BDNF is highly charged with an isoelectric point of 10 and is unstable^90^. For example, only 0.0024% of injected BDNF dose per gram of tissue (% ID/g) was found in the rat hippocampus 60 min after delivery^29^. By contrast, the peak hippocampal BDNF concentration achieved 4 h after i.n. BDNF-CT administration in the present study was more than 400-fold higher (1.00% ID/g or equivalent of 100 ng/g), and above the concentration (20 ng/mL) reported to enhance survival of hippocampal neurons in cell cultures^91,92^ and to induce LTP in hippocampal slices^78^. Further, the peak BDNF-CT hippocampal concentration that we detected is several magnitudes higher than previously reported using other BDNF delivery methods. For example, chitosan-enhanced delivery of BDNF across the nasal barrier achieved 0.0096% ID/g in the rat hippocampus^93^. Of note, after i.n delivery, a BDNF concentration of 0.0057% ID/g (or 4 ng/mL)^29^ upregulated frontal cortex pAkt in rats. In cell cultures, BDNF concentrations (<5 ng/mL) are shown to induce activation of Akt, but not neuroprotection^94^. By contrast, we detected only nonsignificant changes in hippocampal BDNF, Akt, and pAkt levels after daily i.n. delivery of native BDNF for 4 days. The apparent discrepancy between studies could be due to differences in animal models and species, BDNF exposure duration, brain region assessed, and/or BDNF dose.

BDNF-CT increased DG GCL newborn cell survival, proliferation, and neurogenesis in Tat+ mice, compared to saline treated Tat+ controls or Tat- mice. This is consistent with the observed significant increases in mBDNF, Akt, and pAkt levels in BDNF-CT-treated Tat+ mice versus saline treated Tat+ and Tat− mice. Further, these results are consistent with BDNF’s role in neurogenesis^95^ and with previous studies reporting increased numbers of BrdU+ cells following BDNF infusion^30,32,96^ and increased numbers of Ki67+ and DCX+ cells following BDNF lentiviral vector infusions^36^. Importantly, by using noninvasive i.n. BDNF-CT delivery, we were able to achieve results comparable to those achieved in studies of direct hippocampal infusion of BDNF.

Tat+ mice had decreased Ki67+ cell densities compared to Tat− mice. Also, Tat+ mice had impaired hippocampal neurogenesis, as reflected by the decreased percentage of BrdU+ cells that are DCX+ in the GCL. Densities of BrdU+ cells in the GCL were slightly, but not significantly lower in Tat+ vs. Tat− mice, indicating a relatively intact survival of newborn cells after 7 days of Tat induction. Our results are consistent with results reported in previous studies^97–100^. Because previous iTat mouse studies^99^ showed that Tat induced toxic effects on the GLC newborn cell proliferation and differentiation into young neurons, we did not compare Tat versus Dox effects in this study. Tat-induced toxic effects on neurogenesis were completely reversed by BDNF-CT treatment in our study.

Hippocampal SYP and MAP2 immunoreactivities were higher in BDNF-CT treated versus saline treated Tat+ mice, indicating that BDNF-CT treatment enhanced synaptogenesis and dendritic integrity and that these effects were sustained for at least 5 days. These results are consistent with studies documenting increased SYP^101,102^ and MAP2^103^ expressions following BDNF treatment, and with studies reporting protective effects of BDNF against synaptodendritic injury^104^. HIV-1 infection causes synaptodendritic injury, and decreased SYP and MAP2 biomarkers have been associated with higher viral load and impaired cognitive function in HAND^105^. Tat exposure decreased SYP^58,106^ and MAP2^107,108^ in cortical and hippocampal neuronal cultures. Also, Tat injection into the rat hippocampus^109^ and Tat expression in Dox-treated iTat mice^99^ lowered MAP2. Tat decreases SYP and MAP2 levels through mechanisms correlated with BDNF downregulation^58^. BDNF-CT treatment was able to reverse these neurotoxic effects of Tat. Further studies are required to elucidate long-term effects of BDNF-CT on dendritic and/or spine morphology associated with learning and memory in Tat+ mice.

Tat+ mice exhibit learning and memory deficits^59^. As hypothesized, BDNF-CT treatment enhanced spatial learning and memory and cognitive flexibility in Tat+ mice, which are reflected as improved performance in the BMT acquisition and reversal learning phases, respectively. In the reversal learning phase, BDNF-CT-treated mice took less time to find a new escape hole location, used more efficient routes to the escape hole and made fewer reference errors. BDNF-CT treatment increased speed in Tat+ mice only in the acquisition phase, but not in reversal learning phase. This effect was task specific. BDNF-CT did not impair motor functions in the OF test and this finding is consistent with previous preclinical BDNF toxicity studies^110^. Since BDNF-CT treatment was administered only during Tat induction and terminated before behavioral testing, our results indicate that i.n. BDNF-CT treatment exerts durable cognitive-enhancing effects that are sustained for at least 5 days.

Enhanced recognition memory performance also was observed in BDNF-CT-treated Tat+ mice, as reflected by a higher NORT % recognition index (RI). Our findings of a BDNF-CT dose-effect relationship on NORT may indicate that a threshold BDNF dose is necessary for BDNF to act as a cognitive enhancer in Tat+ mice. This underscores the importance of using methods capable of achieving efficient brain BDNF delivery. The BMT and NORT performance deficits rescued by BDNF-CT are consistent with the reported role of BDNF in learning and memory^21–24^. By using noninvasive i.n. BDNF-CT delivery, we were able to achieve results similar to those reported in studies of direct BDNF brain infusion in rodent dementia models^25,111^.

BDNF-CT induced cognitive enhancement is likely due to the molecular changes we detected in BDNF-CT-treated Tat+ mice. These include increased PI3K/Akt pathway signaling, and enhanced SYP and MAP2 expressions. PI3K/Akt pathway activation is directly correlated with spatial memory formation, as antisense BDNF inhibited activation of PI3K/Akt signaling and inhibited spatial learning^112^. Loss of hippocampal SYP^113^ or MAP2^114^ proteins correlates with spatial memory impairments. Tat decreases SYP and MAP2 levels and impairs neurite outgrowth through mechanisms correlated with BDNF downregulation^58^. The SYP and MAP2 increases we observed in the hippocampus of Tat+ mice treated with BDNF-CT correlated with decreased errors and improved BMT reversal learning task performance. Because immature neurons identified by DCX+ staining are not fully incorporated into hippocampal networks, they may not have directly contributed to the positive behavioral outcome after BDNF-CT treatment. However, BDNF-induced neurogenesis, synaptogenesis, and synaptic plasticity is known to have a positive effect on memory^21–24^.

There are limitations to using an HIV-1 iTat mouse model. iTat mice can be induced to express Tat protein but not other HIV viral proteins. Also, due to a leaky transgene promotor, uninduced adult iTat mice express chronic low levels of Tat with aging^115^. Despite these limitations, we were able to document favorable BDNF-CT neurochemical, cellular, and behavioral effects in 10–14-week-old mice exposed to neurotoxic levels of Tat. Further, BDNF-CT-induced changes were independent of Tat levels, as BDNF-CT treatment did not affect hippocampal expression of Tat (Supplementary Fig. 2). Finally, in addition to Tat, other HIV proteins including gp120, gp140, Nef, Vpr, and Rev, exert neurotoxic effects^116^. Future studies will examine BDNF-CT effects in female and male animal models of HIV/neuroAIDS, in which some or all of these proteins are expressed.

The present study represents the first in vivo demonstration of noninvasive clathrin-mediated i.n. delivery of BDNF to the mouse brain resulting in neurorestorative and cognitive-enhancing effects in an animal model of Tat neurotoxicity in HIV/neuroAIDS. These results might also be applicable to other viruses that can induce neuronal damage and cognitive deficits (e.g., COVID-19)^117^. The convergence of molecular, cellular, and behavioral effects of BDNF-CT administration indicates that clathrin NPs enable efficient delivery of BDNF into the brain. Thus, clathrin nanotechnology may be able to enhance neuronal regeneration and plasticity, and may help restore brain function more efficiently than existing treatments. This nanotechnology has the potential to become a powerful tool in regenerative medicine, and in the future may lead to the development of targeted drug delivery systems, and also neuronal protection and repair platforms.

## Methods

### BDNF-CT NP preparation and characterization

Clathrin-coated vesicles (CCVs) were first isolated from pig brains (Pel-Freez Biologicals, Rogers, AR) and clathrin triskelia (CT) (ExQor Tech., Boston, MA) were then isolated from CCVs using published methods^4,118^. CCVs (Fig. 1a) and CT (Fig. 1b) were characterized by Electron Microscopy (1200× Jeol, Tokyo, Japan)^4^. Concentrations of isolated and purified CT were determined using the Bradford Protein Assay Kit (Bio Rad Laboratories, Hercules, CA, 500-0201) and Synergy HT Multi-Detection Reader (BioTek Instruments, Winooski, VT). Maleimide-Polyethylene glycol-*N*-Hydroxysuccinimide (Maleimide-PEG-NHS, MW3500, JenKem Tech, Plano, TX) was used to crosslink recombinant human brain-derived neurotrophic factor (BDNF) (Fisher Scientific, Waltham, MA, 50-721-243) to CT at a 3:1 molar ratio, using published methods^119^. PEGylation of BDNF increases its half-life in the blood and cerebrospinal fluid, and reduces its systemic clearance while minimally affecting biological activity^120^. PEGylated BDNF was reacted with CT in phosphate-buffered saline (PBS, pH 7.4) overnight at 4 °C. The maleimide in PEG chains has high affinity for cysteine sulfhydryl groups. The chemical reactivity of CT’s cysteines was utilized to attach BDNF-PEGs to CT^4^. Resulting BDNF-CT NPs were purified by ultrafiltration using Nanosep 100K OMEGA devices (Pall Life Sciences, Port Washington, NY, OD100C33) to remove unreacted molecules. BDNF Emax ELISA (Promega, Madison, WI) and Bradford assays were performed to calculate final BDNF and CT concentrations. Sodium dodecyl sulfate polyacrylamide gel electrophoresis (SDS-PAGE) was used to assess NP purity and molecular weight increases following conjugations. Dynamic light scattering (DLS) was performed with an LB-550 instrument (Horiba, Kyoto, Japan) to analyze BDNF-CT size and uniformity using published methods^4^.

### Animals and housing

Adult iTat male 10–14-week-old mice^45,46^ were group-housed (*n* = 2–4/cage) in a controlled environment with a 12/12 h light/dark cycle. iTat mice have a doxycycline (Dox)-inducible Tat transgene under the control of an astrocyte-specific glial fibrillary acidic protein promoter, which produces HIV-Tat protein^45,46^. Male mice were selected for NP studies because they are more vulnerable to HIV-1 Tat induced CNS damage than females^45,121^. All mice had ad libitum access to food and water in home cages. Mice were acclimated for a minimum of 1 week prior to initiating procedures. All studies were conducted using procedures approved by the McLean Institutional Animal Care and Use Committee (protocols: 2016N000595 and 2017N000127). All procedures conformed to NIH and National Research Council guidelines on the care and use of laboratory animals.

### Conditional induction of Tat protein in the brain

The Tat induction paradigm was based on previous reports^59^. Briefly, to induce Tat expression (resulting in Tat+ mice), iTat mice were treated once daily with an i.p. 100 mg/kg dose of doxycycline hyclate (Dox; Sigma-Aldrich, St. Lois, MO, D9891) for 4 or 7 days^59^ (experimental designs are provided in Supplementary Table 1.) Dox solution was prepared fresh each day in sterile 0.9% saline and shielded from light. Hippocampal Tat expression was evaluated using Western blotting (Supplementary Figs. 2 and 5).

### Intranasal NP administration

Mice were sedated using 3% (v/v) vaporized isoflurane, and upon loss of consciousness and righting reflexes, mice were placed in a supine position while horizontally maintaining the ventral side of their heads. A total volume of 40 µL of NP solution or saline was administered i.n. to each mouse by micropipette in 5 µL increments, alternating between each naris every 90 s.

Mice took 3–5 min to wake up after completing nasal procedures and were immediately returned to their home cage.

### Radioactive-NP studies

BDNF-CT were radiolabeled with ^[3]^H-NSP (NET632H005MC, MW = 171 Da, PerkinElmer, Shelton, CT) according to a standard labeling method to make ^[3]^H-CT-BDNF NPs^122^. Briefly, the ^[3]^H-NSP solution (0.75 mCi) was placed in a glass tube and solvent was evaporated in a gentle stream of nitrogen for 20 min. The CT protein sample (1 mg, MW = 650 kDa) in 0.5 mL of borate buffer (pH 8) was transferred to the glass tube, incubated with ^[3]^H-NSP, and stirred for 4 h at 4 °C. To remove non-reacted particles, protein was dialyzed in PBS buffer (pH 7.4) by using Biotech CE membranes (100 kDa, Spectrum Laboratories, Rancho Dominguez, CA) until the radioactivity in the last three buffer solutions was at background level. PEGylated BDNF (100 µg of BDNF) was incubated with ^[3]^H-CT in PBS buffer (pH 7.4) overnight at 4 °C. This method was selected to avoid adding additional positive charges to BDNF, and to preserve its function^123^. Resulting ^[3]^H-CT-BDNF NPs were purified by ultrafiltration using Nanosep 100 K OMEGA devices (Pall Life Sciences, OD100C33) as described previously. Radioactivity in the ^[3]^H-CT-BDNF samples was determined using scintillation counting methods, and protein concentration was assessed with Bradford Protein Assay Kit (Bio-Rad Laboratories, 500-0201).

iTat male mice received Dox (100 mg/kg/d, i.p.) for 7 days (Tat+ mice). On day 8th, Tat+ mice were given i.n. ^[3]^H-CT-BDNF. Each mouse received 2.6 µCi of ^[3]^H-CT-BDNF (10 µg BDNF + 30 µg CT). The concentration of ^[3]^H-CT-BDNF was quantitatively assessed in different brain regions of Tat+ mice at 2, 4, and 24 h after intranasal administration. Brains were rapidly removed and dissected. Brain regions (e.g., olfactory bulb, frontal cortex, striatum, hippocampus, hypothalamus, cerebellum, and remaining cortex) were weighed, homogenized, and dissolved in 0.5 mL Solvable (PerkinElmer, 6NE9100) for 4 h at 50 °C. Samples then were decolorized by incubation in 0.1 mL of 30 % H_2_O_2_ for 1 h at 50 °C. Hionic-Fluor (PerkinElmer, 6013311) scintillation cocktail (4 mL) was added to cooled samples. Concentrations were determined based on radioactivity that was measured by standard scintillation counting methods using a Beckman LC6500 scintillation system.

### Fluorescent-NP studies

BDNF-CT brain distribution and TrkB receptor targeting were evaluated using double labeling immunofluorescence (IF) with epifluorescence and confocal microscopy. NHS-Rhodamine (Fisher Scientific, 53031) was conjugated to CT using the manufacturer’s protocol. Rhodamine-labeled CT were then reacted with PEGylated BDNF (see the NP preparation section) to synthesize Rhodamine-labeled BDNF-CT NPs (BDNF-CT-Rho). Non-reacted particles were removed by ultrafiltration using Nanosep 100K OMEGA devices (Pall Life Sciences, OD100C33) and NP specificity was tested in vitro using mouse hippocampal slices. To block BDNF-CT binding to TrkB in brain slices, a 10-fold molar excess of the recombinant human TrkB-IgG chimera (#688-TK, R&D Systems, Minneapolis, MN) was incubated with NPs (Supplementary Fig. 6). For in vivo studies, iTat mice received Dox (100 mg/kg/d) for 7 days. On day 8 mice were given a single dose of i.n. BDNF-CT-Rho (0.3 mg/kg of BDNF and 2.4 mg/kg of CT) or saline (*n* = 2–3/group). After 4 h, mice were anesthetized using pentobarbital (120 mg/kg, i.p.), and then transcardially perfused with 4% paraformaldehyde (PFA). Extracted brains were post-fixed in 4% PFA for 24 h and subsequently cryoprotected with 30% sucrose. Brains were embedded in Tissue-Tek O.C.T. compound (Sakura FineTek, Torrance, CA, 4583) and cryosectioned to obtain 20 µm-thick coronal sections. Sections were slide-mounted and stained as described below (see the “Immunofluorescent staining” section).

### NP delivery into the brain and NP signaling

Tat+ mice were randomized into four i.n. treatment groups: saline (Tat+/Sal), BDNF (0.3 mg/kg; Tat+/BDNF), CT (2.4 mg/kg; Tat+/CT), or BDNF-CT (Tat+/BDNF-CT). During a 4-day Tat induction period, animals (Tat+) received Dox (100 mg/kg/d, i.p.) at 9 am. Nasal treatments were administered daily 5 h after Dox i.p. injections. WB studies were performed after 4 days of Dox induction. The 4-day induction paradigm has been shown to significantly increase brain Tat levels in iTat mice^59^. A separate cohort of non-induced iTat mice (Tat−) that had received saline for 4 days (Tat−/Sal) was included as a global control group. Four hours after the last i.n. treatment, mice were anesthetized using pentobarbital (120 mg/kg, i.p.). Their brains were extracted to assess BDNF levels and NP signaling. Using WB analysis, levels of Tat, mature BDNF (mBDNF), proBDNF, TrkB, phosphorylated TrkB (pTrkB), and downstream molecules protein kinase B (Akt) and phosphorylated Akt (pAkt) were determined.

### Western blot

Hippocampal lysates were prepared^124^ and protein concentrations were determined using the DC™ Protein Assay kit II (Bio Rad Laboratories, 5000112). Proteins were electrophoresed (40 µg/lane) using Mini-Protean TGX Precast Gels (Bio Rad Laboratories, 4569033) and Tris-Glycine SDS buffer (Bio Rad Laboratories, 1610732) at 150 volts for 40 min at 4 °C. Resolved proteins were transferred to polyvinylidene difluoride (PVDF) membranes (Bio Rad Laboratories, 1620177) at 16 V overnight at 4 °C, and blocked in Tris-buffered saline containing 0.05% (w/v) Tween-20 (TBST) and 5% (w/v) dry milk for 1 h at RT. Membranes then were incubated overnight at 4 °C with the following antibodies: anti-BDNF (1:200, Santa Cruz Biotechnology, Dallas, TX, SC-546), or anti-Akt (1:1000, C6E7, Cell Signaling, Danvers, MA, 4691), or anti-pAkt (1:1000, D9E, Cell Signaling, 4060), or anti-TrkB (1:1000, Santa Cruz Biotechnology, SC-8316), or anti-pTrkB/A (1:1000, Cell Signaling, 9141), or anti-Tat^125^ (1:500, NT3, 2D1.1 NIH AIDS Reagent Program, 4138). Membranes were washed and incubated with appropriate horseradish peroxidase-conjugated secondary antibodies (1:5000, Cell Signaling) for 1 h, and developed using Clarity Western ECL Substrates (Bio-Rad Laboratories, 170-5060). Membranes were stripped using Restore Plus WB Stripping Buffer (Fisher Scientific, 46428) and re-probed for β-actin (1:1000, C4, Santa Cruz, SC-47778), which was used as a loading control. Because BDNF increases Akt levels^86^, β-actin was also used as a loading control for pAkt. Images were acquired and analyzed using a Chemi Doc XRS + molecular imager with Image Lab software (ver. 6.0, Bio-Rad Laboratories). WB band intensities were quantified using the Volume Tools in the Image Lab software and normalized to matched β-actin loading controls. Investigators were blinded to treatment conditions. Percent differences from healthy saline-treated Tat− controls were calculated. (WB images are presented in Fig. 3 and Supplementary Figs. 1, 2 and 5. Antibody details are provided in Supplementary Table 2).

### Newborn cell survival, proliferation, and neurogenesis

iTat mice were administered i.p. saline (Tat- controls) or Dox (Tat+) for 7 days to induce Tat expression. Tat+ mice were randomized into two treatment groups and were given either i.n. BDNF (0.3 mg/kg) conjugated to CT (2.4 mg/kg) (Tat+/BDNF-CT), or saline (Tat+/Sal) 5 h after their Dox injections. Tat− mice also received i.n. saline 5 h after i.p. saline injections (Tat−/Sal). BDNF alone and clathrin alone groups were excluded in these experiments, because neither treatment elicited significant molecular changes in the hippocampus in WB experiments. During the first two treatment days, all animals also received bromodeoxyuridine (BrdU, 50 mg/kg, i.p.) at 7 am and 7 pm. On day 7, animals were anesthetized at 7 pm using pentobarbital (120 mg/kg, i.p.), and transcardially perfused with 4% PFA to fix brains. We evaluated doublecortin (DCX), a marker for neurogenesis that was previously observed in nearly all 7-day-old newborn BrdU-positive (BrdU+) cells in mouse hippocampal GLC^126^. Therefore, a 7-day time point was selected to assess BDNF-CT induced neurogenesis. Brains were post-fixed in 4% PFA for 24 h and then cryoprotected with 30% sucrose. Brains were embedded in Tissue-Tek O.C.T. compound (Sakura FineTek, 4583) and stored at −80 °C.

### Immunohistochemistry (IHC) and image analysis

BrdU, Ki67, and DCX were used as indicators of new born cell survival^127^, proliferation^128^, and neurogenesis^129^, respectively. IHC analysis of the hippocampal GCL was performed to determine densities of BrdU-positive (BrdU+), Ki67-positive (Ki67+), and DCX-positive (DCX+) cells. Slide-mounted coronal sections (20 µm thick) were incubated in 10% methanol (v/v) in PBS with 0.3% hydrogen peroxide for 10 min at room temperature (RT). Sections then were incubated at 99 °C for 15 min in the sodium citrate buffer (10 mM, pH 6) for heat-mediated epitope retrieval (HIER), washed and then blocked in 0.2% Triton X-100/PBS (PBST) with 10% normal serum for 20 min at RT. Sections were probed with well-characterized and widely used primary antibodies: anti-BrdU (1:100, BD Biosciences, 347580), or anti-Ki67 (1:200, BD Biosciences, 550609), or anti-DCX (1:100, Santa Cruz, SC-8066) overnight at 4 °C, and then with appropriate biotinylated secondary antibody (1:200–500, Jackson Immuno Research Labs, West Grove, PA) for 30 min at room temperature (RT). The specificity of IHC test was verified by primary antibody omission or inclusion of a non-immune antibody of the same isotype and at the same concentration as the primary antibody. The Vectastain ABC Elite detection system (Vector Labs, Burlingame, CA, PK-7100) was used to visualize staining following manufacturer-recommended procedures. Briefly, sections were incubated for 30 min at RT in avidin–biotin–HRP complex (ABC reagent), and peroxidase activity was detected by incubation with 3,3′ diaminobenzidine (DAB) with nickel for 3 min. Sections were counterstained with hematoxylin (Sigma-Aldrich, MHS16) for 30 s, washed, dehydrated in graded ethanols, cleared and cover-slipped. (Images were presented in Fig. 4). An investigator blinded to treatment conditions determined the number of positively labeled cells by stereological counting of every 6th section spanning the entire hippocampus, using the Stereo Investigator 11 (MBF Bioscience, Williston, VT) coupled with Zeiss Axio Scope A2 microscope (Zeiss, Thornwood, NY). Settings for gain, aperture, contrast, and brightness were held constant for the analysis of a series of matching sections from the same rostral**-**caudal level. For each section, GCL boundaries were outlined and the surface area was electronically measured and recorded. Hippocampal GCL surface area was multiplied by tissue thickness to calculate volumes and to determine cell densities.

### Immunofluorescent (IF) staining

Slide-mounted hippocampal coronal sections underwent HIER treatment for 1 h in citric acid (pH 6) at 99 °C. Sections were then cooled to RT, permeabilized, blocked, and incubated with the following primary antibodies: anti-TrkB antibody (1:200, H181, Santa Cruz, SC-8316); anti-BrdU (1:100, B44 BD Biosciences, C:347680); anti-DCX (1:250, C-18, Santa Cruz, SC-8066-R)**;** anti-SYP (1:500, D-4 Santa Cruz, SC-17750) and anti-MAP2 (1:500, Cell Signaling, 4542S) for 24 h at 4 °C. Sections were then incubated with appropriate secondary antibodies: goat anti-mouse Alexa Fluor 568 (1:200, Fisher Scientific, A-11004) and/or goat anti-rabbit Alexa Fluor 488 (1:200, Fisher Scientific, A-11008) for 2 h at RT. The specificity of IF test was verified by primary antibody omission or inclusion of a non-immune antibody of the same isotype and at the same concentration as the primary antibody. Slides were then cover-slipped with Vectashield Mounting Medium with DAPI (Vector Labs, H-1200).

### Image acquisition

Brain sections were imaged with a confocal microscope (Leica Microsystems, Buffalo Grove, IL, TCS SP8). Multiple optical sections spanning 20 µm in the z-dimension were acquired in 1 µm steps at 63× (oil immersion objective). Rhodamine was excited with 561 nm laser and collected at 575 nm emission. Alexa 568 was excited with 578 nm laser and collected at 603 nm emission. Alexa Fluor 488 was excited at 488 nm and collected at 519 nm emission. The Leica Application Suite X (Leica Microsystems) was used to merge images and assess co-labeling. Images were also acquired using 10×/0.3 Ph1, 20×/0.5 Ph2 and 40×/0.75 Ph2 EC Plan-NEOFLUAR M27 objectives with ORCA-ER C4742-80 CCD camera (Hamamatsu, Japan) on the Zeiss A1 microscope (Carl Zeiss Inc.) with Micro-Manager software (v1.2, NIH). For LED illumination (100%), 365, 470, and 540–580 nm LED modules (Carl Zeiss Inc.) were used for DAPI, Alexa Fluor 488 and Alexa Fluor 568, respectively. Settings for gain, aperture, contrast, and brightness were held constant throughout the study. An investigator blinded to treatment conditions analyzed every 6th hippocampal section from the same rostral**-**caudal level using an image analysis system (Fiji, v1.52H, NIH). Anatomical regions and landmarks were obtained from the Allen Mouse Brain Atlas (https://atlas.brain-map.org).

### Analysis of BrdU and DCX markers

Twenty-five BrdU+ cells in the GCL were randomly selected per mouse (*n* = 3/group) based on published protocols^126^, and assessed for co-localization with DCX marker using NIH image analysis system (Fiji, v1.52H, NIH). Briefly, BrdU+ cells were identified in images by processing the red channel in the following manner: background subtraction (Rolling Ball 2000 px); variance (5 px) and mean filters (2 px); and a binary image was generated using Renyi’s entropy threshold. Finally, particle analysis was used to generate masks that were applied to the raw images. BrdU+ cells in the GLC and within two cell body widths of the GCL in the subgranular zone were automatically counted in every 6th section spanning the entire mouse hippocampus. Twenty-five BrdU+ cells were then randomly selected per animal from this pool of BrdU+ cells using a random number generator. These randomly selected BrdU+ cells were visually inspected, images were pseudo-colored red and green and merged, and cells positive for both the BrdU+ and DCX markers were then counted. The investigator who performed these assessments was blinded to treatment conditions. A confocal microscope (Leica Microsystems, Buffalo Grove, IL, TCS SP8) was used to image and verify double labeled cells. Double-labeled cells were expressed as the percentage of BrdU+ cells. The mean percentages were calculated for treatment groups and compared using ANOVA. (Images were presented in Fig. 5 and Supplementary Fig. 7).

### Analysis of SYP and MAP2 markers

SYP^130^ is a marker of synaptogenesis and MAP2 is a marker of dendritic integrity^131^. These markers were evaluated in a cohort of mice (*n* = 6–8/group) that completed cognitive tests by using NIH image analysis system (Fiji, v1.52H, NIH). Every 6th (20 µm thick) hippocampal section was analyzed. Each image represented a randomly selected field of view of 12-bit depth 1344 × 1024 pixel (*x*–*y* plane) of the hippocampal region of interest (ROI) (e.g., DG, CA1, or CA3) that was consistent across animals. Approximately ten images (ten half-sections) per animal were analyzed for each ROI. The automated Otsu’s method was applied to segment the image and find the optimal threshold value of the image by maximizing the weighted between-class variance (e.g., foreground vs. background) and minimizing within-class variance. This method ensures that the area analyzed corresponds to the immunomarker of interest. The automated ImageJ script was then used to analyze segmented images and to measure the mean pixel intensity and area of pixels covered by the immunoreactive staining. The mean intesities were calculated for the hippocampal brain regions (e.g., DG, CA1, and CA3) for each animal and compared between BDNF-CT and saline treated groups using *t*-tests. (Images were presented in Fig. 6).

### Behavioral testing of learning and memory

During a randomized placebo-controlled preclinical study, animals were videotaped using ANY-maze software (Stoelting Co., Wood Dale, IL), and data were analyzed by investigators blinded to treatment conditions. Effects of NP treatments on hippocampal-dependent learning and memory were examined using the novel object recognition test (NORT) and Barnes maze test (BMT). These tests have been successfully used in iTat mice to show significant differences in learning and memory between Tat+ and Tat− mice^59^ and test procedures were adapted from that study. Tat+ mice were excluded from studies for any one of the following reasons: death, major trauma, anatomical brain and/or other organ malformations, or serious Tat-induced medical problems (e.g., seizures, difficulties breathing, severe gastrointestinal problems, non-healing wounds etc.).

### Barnes Maze test

iTat mice were treated for 7 days with Dox (100 mg/kg/d, i.p.) and randomized into two groups: a study group (*n* = 14) and a control group (*n* = 16). Four mice were excluded from studies for the reasons listed above. Daily Dox treatments were followed 5 h later by i.n. administration of saline or BDNF-CT NPs (the mean BDNF dose in NPs was 0.23 mg/kg). Following completion of treatments, the BMT (San Diego Instruments, CA) was used to assess spatial learning and memory^59^. Testing was carried out during an acquisition (test days 1–4) and a reversal learning (on test day 5) phase. Before testing began mice were habituated to the BMT apparatus by allowing free exploration for 1 min (without allowing escape), and then by placing them into the unattached escape chamber for 30 s. During the acquisition phase, mice were trained to find and enter the escape chamber during two 3-min acquisition trials, with a 15 min inter-trial interval (ITI). Mice that failed to enter the escape chamber by the end of each trial were gently guided to enter the chamber. Bright light and static radio noise were used to motivate mice to escape the maze. These stimuli were terminated once mice entered the escape chamber. During the reversal learning phase, cognitive flexibility was tested by relocating the escape chamber 150° counterclockwise from its original location and conducting four 3 min trials with 15 min ITIs. All trials were videotaped and analyzed using ANY-maze™ software (Stoelting Co., Wood Dale, IL) to quantify escape latencies, numbers of reference errors, path efficiency and average speed. Path efficiency is calculated by dividing the straight-line distance between the starting and finishing position by the total distance traveled by the animal during a trial. A mouse was determined to have escaped when 100% of his body and all four paws entered the escape chamber. Head deflections into non-escape holes were counted as errors. The maze and the escape chamber were cleaned with 70% ethanol between trials to remove residual secretions and odors.

### Novel object recognition test

NORT, a test based on a mouse’s natural tendency to explore their environment, was used to test effects of treatments on recognition memory^59,132^. The NORT consisted of three 10 min phases separated by 10 min ITIs. During each phase, animals freely explored objects placed in rectangular test chambers. For phase 1, two identical objects (e.g., dice) were placed at opposite ends of cages on centerlines, 2 cm away from opposite end walls. During phase 2, the location of one object was moved laterally closer to a side wall. In phase 3, one of the original objects was replaced with a novel object (e.g., a marble), and both objects were placed on the same centerline locations used in phase 1. Phases were videotaped using ANY-maze software (Stoelting Co., Wood Dale, IL) and were analyzed by a blinded observer for time spent on each object, which was defined as physical contact with the object (except via the tail), or sniffing or other manipulation of the object. Data were converted to % recognition index (RI) using the following formula: % RI = (time spent on novel object / time spent on both objects) × 100. Objects were thoroughly cleaned with 70% ethanol between phases and subjects to remove secretions and odors. NORT was conducted at the end of the second day of the BMT.

### Evaluation of BDNF-CT toxic effects on motor functions

To evaluate potential BDNF-CT toxic effects on mouse locomotor activity we tested wild type mice using Open Field (OF) test. C57BL/6J male mice received BDNF-CT (*n* = 10) or saline (*n* = 10) i.n. daily for 7 days and were tested with OF on day 7. Mice were placed in the lower left corner of a square Plexiglas box (40 × 40 × 35 cm; Stoelting Co., Wood Dale, IL) and allowed to explore it for 20 min. Movement was monitored, recorded and digitally encoded by an ANY-maze software. The average speed and total distance traveled were used as indices of locomotor behavior.

### Statistics and reproducibility

WB and IHC data were analyzed using Prism 7 (GraphPad Software Inc., La Jolla, CA). Behavioral data were analyzed using the JMP Pro 14 software package (SAS Institute Inc., Carey, NC). Data values that were two standard deviations or more away from group means were a priori defined as outliers and excluded from analyses. Significance was defined as *P* < 0.05. One-way analysis of variance (ANOVA) followed by Tukey–Kramer post hoc testing was used to assess WB and IHC group differences. For SYP and MAP 2 analyses treatment groups were compared using *t*-tests. For the BMT, overall differences in latency, numbers of reference errors, path efficiency, and average speed were analyzed via a widely used linear mixed-model repeated measure analysis. Akaike information criterion (AIC) was used to assess model fit^133^ and the model with the lowest AIC value was selected. For BMT the model included the following fixed effects: day (or trial), treatment, and day (or trial) × treatment. RIs from NORT Phase 3 were analyzed for treatment effects using One-Way ANOVA followed by Tukey–Kramer post hoc tests. Correlations between BMT cognitive performance variables and histological quantifications in the hippocampal regions were performed using Pearson product-moment correlations. (Statistical details are provided in Supplementary Tables 3 and 4 and the source data were provided in Supplementary Data 1 file).

### Reporting summary

Further information on research design is available in the Nature Research Reporting Summary linked to this article.

## Supplementary information

Supplementary Information

Description of Additional Supplementary Files

Supplementary Data 1

Reporting Summary

## Data Availability

The source data can be found in the Supplementary Data 1. Other data that support the findings of this study are available from ExQor Technology Inc. These data are available from the authors upon reasonable request and with ExQor Technologies permission.

## References

[1] Illum L (2012). Nasal drug delivery—recent developments and future prospects. J. Control Release.

[2] Gomez D, Martinez JA, Hanson LR, Frey WH, Toth CC (2012). Intranasal treatment of neurodegenerative diseases and stroke. Front. Biosci..

[3] Zhu J, Jiang Y, Xu G, Liu X (2012). Intranasal administration: a potential solution for cross-BBB delivering neurotrophic factors. Histol. Histopathol..

[4] Vitaliano GD, Vitaliano F, Rios JD, Renshaw PF, Teicher MH (2012). New clathrin-based nanoplatforms for magnetic resonance imaging. PLoS ONE.

[5] Brodsky FM (2012). Diversity of clathrin function: new tricks for an old protein. Annu. Rev. Cell Dev. Biol..

[6] Kirchhausen T, Owen D, Harrison SC (2014). Molecular structure, function, and dynamics of clathrin-mediated membrane traffic. Cold Spring Harb. Perspect. Biol..

[7] Preston JE, Joan Abbott N, Begley DJ (2014). Transcytosis of macromolecules at the blood-brain barrier. Adv. Pharmacol..

[8] Granseth B, Odermatt B, Royle SJ, Lagnado L (2007). Clathrin-mediated endocytosis: the physiological mechanism of vesicle retrieval at hippocampal synapses. J. Physiol..

[9] Mills IG (2007). The interplay between clathrin-coated vesicles and cell signalling. Semin. Cell Dev. Biol..

[10] Royle SJ (2006). The cellular functions of clathrin. Cell. Mol. Life Sci..

[11] Baba T (2001). Clathrin-dependent and clathrin-independent endocytosis are differentially sensitive to insertion of poly (ethylene glycol)-derivatized cholesterol in the plasma membrane. Traffic.

[12] Schmid SL, Matsumoto AK, Rothman JE (1982). A domain of clathrin that forms coats. Proc. Natl Acad. Sci. USA.

[13] Bartus, R. T. & Johnson, E. M., Jr. Clinical tests of neurotrophic factors for human neurodegenerative diseases, part 1: Where have we been and what have we learned? *Neurobiol. Dis.* **97**, 156–168 (2017). 10.1016/j.nbd.2016.03.027 27063798

[14] Lu B, Nagappan G, Guan X, Nathan PJ, Wren P (2013). BDNF-based synaptic repair as a disease-modifying strategy for neurodegenerative diseases. Nat. Rev. Neurosci..

[15] Ghosh A, Carnahan J, Greenberg ME (1994). Requirement for BDNF in activity-dependent survival of cortical neurons. Science.

[16] Horch HW, Katz LC (2002). BDNF release from single cells elicits local dendritic growth in nearby neurons. Nat. Neurosci..

[17] Zagrebelsky M, Korte M (2014). Form follows function: BDNF and its involvement in sculpting the function and structure of synapses. Neuropharmacology.

[18] Mattson MP, Maudsley S, Martin B (2004). BDNF and 5-HT: a dynamic duo in age-related neuronal plasticity and neurodegenerative disorders. Trends Neurosci..

[19] Leal G, Afonso PM, Salazar IL, Duarte CB (2015). Regulation of hippocampal synaptic plasticity by BDNF. Brain Res..

[20] Song M, Martinowich K, Lee FS (2017). BDNF at the synapse: why location matters. Mol. Psychiatry.

[21] Yamada K, Mizuno M, Nabeshima T (2002). Role for brain-derived neurotrophic factor in learning and memory. Life Sci..

[22] Cunha C, Brambilla R, Thomas KL (2010). A simple role for BDNF in learning and memory?. Front. Mol. Neurosci..

[23] Bekinschtein P, Cammarota M, Medina JH (2014). BDNF and memory processing. Neuropharmacology.

[24] Andero R, Choi DC, Ressler KJ (2014). BDNF-TrkB receptor regulation of distributed adult neural plasticity, memory formation, and psychiatric disorders. Prog. Mol. Biol. Transl. Sci..

[25] Nagahara AH (2009). Neuroprotective effects of brain-derived neurotrophic factor in rodent and primate models of Alzheimer’s disease. Nat. Med..

[26] Nagahara AH, Tuszynski MH (2011). Potential therapeutic uses of BDNF in neurological and psychiatric disorders. Nat. Rev. Drug Discov..

[27] Poduslo JF, Curran GL (1996). Permeability at the blood-brain and blood-nerve barriers of the neurotrophic factors: NGF, CNTF, NT-3, BDNF. Brain Res. Mol. Brain Res..

[28] Pardridge WM, Kang YS, Buciak JL (1994). Transport of human recombinant brain-derived neurotrophic factor (BDNF) through the rat blood-brain barrier in vivo using vector-mediated peptide drug delivery. Pharm. Res..

[29] Alcala-Barraza SR (2010). Intranasal delivery of neurotrophic factors BDNF, CNTF, EPO, and NT-4 to the CNS. J. Drug Target..

[30] Scharfman H (2005). Increased neurogenesis and the ectopic granule cells after intrahippocampal BDNF infusion in adult rats. Exp. Neurol..

[31] Bekinschtein P (2008). BDNF is essential to promote persistence of long-term memory storage. Proc. Natl Acad. Sci. USA.

[32] Kuipers SD (2016). BDNF-induced LTP is associated with rapid Arc/Arg3.1-dependent enhancement in adult hippocampal neurogenesis. Sci. Rep..

[33] Blurton-Jones M (2009). Neural stem cells improve cognition via BDNF in a transgenic model of Alzheimer disease. Proc. Natl Acad. Sci. USA.

[34] Zhang W (2014). Neural stem cell transplants improve cognitive function without altering amyloid pathology in an APP/PS1 double transgenic model of Alzheimer’s disease. Mol. Neurobiol..

[35] Ando S (2002). Animal model of dementia induced by entorhinal synaptic damage and partial restoration of cognitive deficits by BDNF and carnitine. J. Neurosci. Res..

[36] Quesseveur G (2013). BDNF overexpression in mouse hippocampal astrocytes promotes local neurogenesis and elicits anxiolytic-like activities. Transl. Psychiatry.

[37] Sirianni RW, Olausson P, Chiu AS, Taylor JR, Saltzman WM (2010). The behavioral and biochemical effects of BDNF containing polymers implanted in the hippocampus of rats. Brain Res..

[38] Angelov B (2014). Multicompartment lipid cubic nanoparticles with high protein upload: millisecond dynamics of formation. ACS Nano.

[39] Chen H (2016). Focused ultrasound-enhanced intranasal brain delivery of brain-derived neurotrophic factor. Sci. Rep..

[40] Geral C, Angelova A, Lesieur S (2013). From molecular to nanotechnology strategies for delivery of neurotrophins: emphasis on brain-derived neurotrophic factor (BDNF). Pharmaceutics.

[41] Zhang Y, Pardridge WM (2006). Blood-brain barrier targeting of BDNF improves motor function in rats with middle cerebral artery occlusion. Brain Res..

[42] Khalin I (2016). Brain-derived neurotrophic factor delivered to the brain using poly (lactide-co-glycolide) nanoparticles improves neurological and cognitive outcome in mice with traumatic brain injury. Drug Deliv..

[43] Jiang, Y. et al. Nanoformulation of brain-derived neurotrophic factor with target receptor-triggered-release in the central nervous system. *Adv. Funct. Mater.* **28**, 1703982 (2018). 10.1002/adfm.201703982 PMC5958903 29785179

[44] Ma XC (2016). Intranasal delivery of recombinant AAV containing BDNF fused with HA2TAT: a potential promising therapy strategy for major depressive disorder. Sci. Rep..

[45] Kim BO (2003). Neuropathologies in transgenic mice expressing human immunodeficiency virus type 1 Tat protein under the regulation of the astrocyte-specific glial fibrillary acidic protein promoter and doxycycline. Am. J. Pathol..

[46] Langford D (2018). Doxycycline-inducible and astrocyte-specific HIV-1 Tat transgenic mice (iTat) as an HIV/neuroAIDS model. J. Neurovirol..

[47] Mocchetti I, Bachis A, Campbell LA, Avdoshina V (2014). Implementing neuronal plasticity in NeuroAIDS: the experience of brain-derived neurotrophic factor and other neurotrophic factors. J. NeuroImmune Pharmacol..

[48] Bachis A, Avdoshina V, Zecca L, Parsadanian M, Mocchetti I (2012). Human immunodeficiency virus type 1 alters brain-derived neurotrophic factor processing in neurons. J. Neurosci..

[49] Albrecht D (2006). Trophic factors in cerebrospinal fluid and spinal cord of patients with tropical spastic paraparesis, HIV, and Creutzfeldt-Jakob disease. AIDS Res. Hum. Retroviruses.

[50] Meeker RB, Poulton W, Markovic-Plese S, Hall C, Robertson K (2011). Protein changes in CSF of HIV-infected patients: evidence for loss of neuroprotection. J. Neurovirol..

[51] Fields J (2015). HIV-1 Tat alters neuronal autophagy by modulating autophagosome fusion to the lysosome: implications for HIV-associated neurocognitive disorders. J. Neurosci..

[52] King JE, Eugenin EA, Buckner CM, Berman JW (2006). HIV tat and neurotoxicity. Microbes Infect..

[53] Hudson L (2000). Detection of the human immunodeficiency virus regulatory protein tat in CNS tissues. J. Neurovirol..

[54] Henderson LJ (2019). Presence of Tat and transactivation response element in spinal fluid despite antiretroviral therapy. Aids.

[55] Jones M, Olafson K, Del Bigio MR, Peeling J, Nath A (1998). Intraventricular injection of human immunodeficiency virus type 1 (HIV-1) tat protein causes inflammation, gliosis, apoptosis, and ventricular enlargement. J. Neuropathol. Exp. Neurol..

[56] McLaughlin JP (2017). Conditional human immunodeficiency virus transactivator of transcription protein expression induces depression-like effects and oxidative stress. Biol. Psychiatry Cogn Neurosci Neuroimaging.

[57] Carey AN (2013). Conditional Tat protein expression in the GT-tg bigenic mouse brain induces gray matter density reductions. Prog. Neuro-Psychopharmacol..

[58] Rahimian P, He JJ (2016). HIV-1 Tat-shortened neurite outgrowth through regulation of microRNA-132 and its target gene expression. J. Neuroinflamm..

[59] Carey AN, Sypek EI, Singh HD, Kaufman MJ, McLaughlin JP (2012). Expression of HIV-Tat protein is associated with learning and memory deficits in the mouse. Behav. Brain Res..

[60] Marks WD (2016). HIV-1 Tat causes cognitive deficits and selective loss of parvalbumin, somatostatin, and neuronal nitric oxide synthase expressing hippocampal CA1 interneuron subpopulations. J. Neurovirol..

[61] Fitting S (2013). Synaptic dysfunction in the hippocampus accompanies learning and memory deficits in human immunodeficiency virus type-1 Tat transgenic mice. Biol. Psychiatry.

[62] Ramirez SH (2001). Neurotrophins prevent HIV Tat-induced neuronal apoptosis via a nuclear factor-kappaB (NF-kappaB)-dependent mechanism. J. Neurochem..

[63] Fujimura RK (1997). HIV-1 proviral DNA load across neuroanatomic regions of individuals with evidence for HIV-1-associated dementia. JAIDS.

[64] Wiley CA (1998). Distribution of brain HIV load in AIDS. Brain Pathol..

[65] Anthony IC, Ramage SN, Carnie FW, Simmonds P, Bell JE (2005). Influence of HAART on HIV-related CNS disease and neuroinflammation. J. Neuropathol. Exp. Neurol..

[66] Nir TM (2021). Association of immunosuppression and viral load with subcortical brain volume in an International sample of people living With HIV. JAMA Netw. Open.

[67] Ferguson ML (2006). Conformation of a clathrin triskelion in solution. Biochemistry.

[68] Kocsis E, Trus BL, Steer CJ, Bisher ME, Steven AC (1991). Image averaging of flexible fibrous macromolecules: the clathrin triskelion has an elastic proximal segment. J. Struct. Biol..

[69] Kirchhausen T, Harrison SC, Heuser J (1986). Configuration of clathrin trimers: evidence from electron microscopy. J. Ultrastruct. Mol. Struct. Res..

[70] Kotova, S. et al. AFM visualization of clathrin triskelia under fluid and in air. *FEBS Lett*. **584**, 44–48 (2010). 10.1016/j.febslet.2009.11.039 PMC2801348 19925798

[71] Fitting S (2010). Interactive comorbidity between opioid drug abuse and HIV-1 Tat: chronic exposure augments spine loss and sublethal dendritic pathology in striatal neurons. Am. J. Pathol..

[72] Lee R, Kermani P, Teng KK, Hempstead BL (2001). Regulation of cell survival by secreted proneurotrophins. Science.

[73] Yasuda M (2007). Robust stimulation of TrkB induces delayed increases in BDNF and Arc mRNA expressions in cultured rat cortical neurons via distinct mechanisms. J. Neurochem..

[74] Yang J (2014). proBDNF negatively regulates neuronal remodeling, synaptic transmission, and synaptic plasticity in hippocampus. Cell Rep..

[75] Guo W, Nagappan G, Lu B (2018). Differential effects of transient and sustained activation of BDNF-TrkB signaling. Dev. Neurobiol..

[76] Panja D, Bramham CR (2014). BDNF mechanisms in late LTP formation: a synthesis and breakdown. Neuropharmacology.

[77] Patterson SL (1996). Recombinant BDNF rescues deficits in basal synaptic transmission and hippocampal LTP in BDNF knockout mice. Neuron.

[78] Kang H, Schuman EM (1995). Long-lasting neurotrophin-induced enhancement of synaptic transmission in the adult hippocampus. Science.

[79] Rossi C (2006). Brain-derived neurotrophic factor (BDNF) is required for the enhancement of hippocampal neurogenesis following environmental enrichment. Eur. J. Neurosci..

[80] Woo NH (2005). Activation of p75NTR by proBDNF facilitates hippocampal long-term depression. Nat. Neurosci..

[81] Teng HK (2005). ProBDNF induces neuronal apoptosis via activation of a receptor complex of p75NTR and sortilin. J. Neurosci..

[82] Barnes P, Thomas KL (2008). Proteolysis of proBDNF is a key regulator in the formation of memory. PLoS ONE.

[83] Qiao H, An SC, Xu C, Ma XM (2017). Role of proBDNF and BDNF in dendritic spine plasticity and depressive-like behaviors induced by an animal model of depression. Brain Res..

[84] Numakawa T (2010). BDNF function and intracellular signaling in neurons. Histol. Histopathol..

[85] Brunet A, Datta SR, Greenberg ME (2001). Transcription-dependent and -independent control of neuronal survival by the PI3K-Akt signaling pathway. Curr. Opin. Neurobiol..

[86] Yang JW (2015). BDNF promotes the growth of human neurons through crosstalk with the Wnt/beta-catenin signaling pathway via GSK-3beta. Neuropeptides.

[87] Green MV, Thayer SA (2016). NMDARs adapt to neurotoxic HIV protein Tat downstream of a GluN2A-ubiquitin ligase signaling pathway. J. Neurosci..

[88] Fassnacht M (2005). AKT is highly phosphorylated in pheochromocytomas but not in benign adrenocortical tumors. J. Clin. Endocrinol. Metab..

[89] Yan Q (1997). Expression of brain-derived neurotrophic factor protein in the adult rat central nervous system. Neuroscience.

[90] Barde YA, Edgar D, Thoenen H (1982). Purification of a new neurotrophic factor from mammalian brain. EMBO J..

[91] Lindholm D, Carroll P, Tzimagiorgis G, Thoenen H (1996). Autocrine-paracrine regulation of hippocampal neuron survival by IGF-1 and the neurotrophins BDNF, NT-3 and NT-4. Eur. J. Neurosci..

[92] Johnson-Farley NN, Travkina T, Cowen DS (2006). Cumulative activation of akt and consequent inhibition of glycogen synthase kinase-3 by brain-derived neurotrophic factor and insulin-like growth factor-1 in cultured hippocampal neurons. J. Pharmacol. Exp. Ther..

[93] Vaka SR, Murthy SN, Balaji A, Repka MA (2012). Delivery of brain-derived neurotrophic factor via nose-to-brain pathway. Pharm. Res..

[94] Zheng F, Soellner D, Nunez J, Wang H (2008). The basal level of intracellular calcium gates the activation of phosphoinositide 3-kinase-Akt signaling by brain-derived neurotrophic factor in cortical neurons. J. Neurochem..

[95] Numakawa, T., Odaka, H. & Adachi, N. Actions of brain-derived neurotrophin factor in the neurogenesis and neuronal function, and its involvement in the pathophysiology of brain diseases. *Int. J. Mol. Sci.* **19**, 3650 (2018). 10.3390/ijms19113650 PMC6274766 30463271

[96] Lian D (2016). Exogenous BDNF increases neurogenesis in the hippocampus in experimental Streptococcus pneumoniae meningitis. J. Neuroimmunol..

[97] Fatima M (2016). Tripartite containing motif 32 modulates proliferation of human neural precursor cells in HIV-1 neurodegeneration. Cell Death Differ..

[98] Mishra M, Taneja M, Malik S, Khalique H, Seth P (2010). Human immunodeficiency virus type 1 Tat modulates proliferation and differentiation of human neural precursor cells: implication in NeuroAIDS. J. Neurovirol..

[99] Fan Y, Gao X, Chen J, Liu Y, He JJ (2016). HIV Tat impairs neurogenesis through functioning as a notch ligand and activation of notch signaling pathway. J. Neurosci..

[100] Hill JD, Zuluaga-Ramirez V, Gajghate S, Winfield M, Persidsky Y (2019). Chronic intrahippocampal infusion of HIV-1 neurotoxic proteins: a novel mouse model of HIV-1 associated inflammation and neural stem cell dysfunction. J. NeuroImmune Pharmacol..

[101] Coffey ET, Akerman KE, Courtney MJ (1997). Brain derived neurotrophic factor induces a rapid upregulation of synaptophysin and tau proteins via the neurotrophin receptor TrkB in rat cerebellar granule cells. Neurosci. Lett..

[102] Bamji SX, Rico B, Kimes N, Reichardt LF (2006). BDNF mobilizes synaptic vesicles and enhances synapse formation by disrupting cadherin-beta-catenin interactions. J. Cell Biol..

[103] Fukumitsu H, Ohashi A, Nitta A, Nomoto H, Furukawa S (1997). BDNF and NT-3 modulate expression and threonine phosphorylation of microtubule-associated protein 2 analogues, and alter their distribution in the developing rat cerebral cortex. Neurosci. Lett..

[104] Melo CV (2013). Spatiotemporal resolution of BDNF neuroprotection against glutamate excitotoxicity in cultured hippocampal neurons. Neuroscience.

[105] Levine AJ (2016). Multilevel analysis of neuropathogenesis of neurocognitive impairment in HIV. J. Neurovirol..

[106] Shin AH, Thayer SA (2013). Human immunodeficiency virus-1 protein Tat induces excitotoxic loss of presynaptic terminals in hippocampal cultures. Mol. Cell. Neurosci..

[107] Butler TR, Smith KJ, Self RL, Braden BB, Prendergast MA (2011). Neurodegenerative effects of recombinant HIV-1 Tat(1–86) are associated with inhibition of microtubule formation and oxidative stress-related reductions in microtubule-associated protein-2(a,b). Neurochem. Res..

[108] Aprea S (2006). Tubulin-mediated binding of human immunodeficiency virus-1 Tat to the cytoskeleton causes proteasomal-dependent degradation of microtubule-associated protein 2 and neuronal damage. J. Neurosci..

[109] Maragos WF (2003). Neuronal injury in hippocampus with human immunodeficiency virus transactivating protein, Tat. Neuroscience.

[110] LaBel, C. P. & Foss, J. Use of a rodent neurotoxicity screening battery in the preclinical safety assessment of recombinant-methionyl human brain-derived neurotrophic factor. *Neurotoxicology* **17**, 851–864 (1996). 9086509

[111] Zhang L (2015). Brain-derived neurotrophic factor ameliorates learning deficits in a rat model of Alzheimer’s disease induced by abeta1–42. PLoS ONE.

[112] Mizuno M (2003). Phosphatidylinositol 3-kinase: a molecule mediating BDNF-dependent spatial memory formation. Mol. Psychiatry.

[113] Schmitt U, Tanimoto N, Seeliger M, Schaeffel F, Leube RE (2009). Detection of behavioral alterations and learning deficits in mice lacking synaptophysin. Neuroscience.

[114] Khuchua Z (2003). Deletion of the N-terminus of murine map2 by gene targeting disrupts hippocampal ca1 neuron architecture and alters contextual memory. Neuroscience.

[115] Dickens AM (2017). Chronic low-level expression of HIV-1 Tat promotes a neurodegenerative phenotype with aging. Sci. Rep..

[116] Jaeger LB, Nath A (2012). Modeling HIV-associated neurocognitive disorders in mice: new approaches in the changing face of HIV neuropathogenesis. Dis. Models Mech..

[117] Paterson, R. W. et al. The emerging spectrum of COVID-19 neurology: clinical, radiological and laboratory findings. *Brain* **143**, 3104–3120 (2020). 10.1093/brain/awaa240 PMC7454352 32637987

[118] Zhu Y, Drake MT, Kornfeld S (2001). Adaptor protein 1-dependent clathrin coat assembly on synthetic liposomes and Golgi membranes. Methods Enzymol..

[119] Soderquist RG (2009). PEGylation of brain-derived neurotrophic factor for preserved biological activity and enhanced spinal cord distribution. J. Biomed. Mater. Res. Part A.

[120] Sakane T, Pardridge WM (1997). Carboxyl-directed pegylation of brain-derived neurotrophic factor markedly reduces systemic clearance with minimal loss of biologic activity. Pharm. Res..

[121] Hahn, Y. K. et al. Effects of chronic HIV-1 Tat exposure in the CNS: heightened vulnerability of males versus females to changes in cell numbers, synaptic integrity, and behavior. *Brain Struct. Funct.* **220**, 605–623 (2013). 10.1007/s00429-013-0676-6 PMC4341022 24352707

[122] Boado RJ, Zhang Y, Zhang Y, Pardridge WM (2007). Genetic engineering, expression, and activity of a fusion protein of a human neurotrophin and a molecular Trojan horse for delivery across the human blood-brain barrier. Biotechnol. Bioeng..

[123] Kummer U (1986). Tritium radiolabeling of antibodies to high specific activity with N-succinimidyl [2,3-3H]propionate: use in detecting and analyzing monoclonal antibodies. Methods Enzymol..

[124] Chartoff EH, Mague SD, Barhight MF, Smith AM, Carlezon WA (2006). Behavioral and molecular effects of dopamine D1 receptor stimulation during naloxone-precipitated morphine withdrawal. J. Neurosci..

[125] Dingwall C (1989). Human immunodeficiency virus 1 tat protein binds trans-activation-responsive region (TAR) RNA in vitro. Proc. Natl Acad. Sci. USA.

[126] Snyder JS (2009). Adult-born hippocampal neurons are more numerous, faster maturing, and more involved in behavior in rats than in mice. J. Neurosci..

[127] Wojtowicz JM, Kee N (2006). BrdU assay for neurogenesis in rodents. Nat. Protoc..

[128] Scholzen T, Gerdes J (2000). The Ki-67 protein: from the known and the unknown. J. Cell. Physiol..

[129] Couillard-Despres S (2005). Doublecortin expression levels in adult brain reflect neurogenesis. Eur. J. Neurosci..

[130] Knaus P, Betz H, Rehm H (1986). Expression of synaptophysin during postnatal development of the mouse brain. J. Neurochem..

[131] Caceres A, Banker GA, Binder L (1986). Immunocytochemical localization of tubulin and microtubule-associated protein 2 during the development of hippocampal neurons in culture. J. Neurosci..

[132] Antunes M, Biala G (2012). The novel object recognition memory: neurobiology, test procedure, and its modifications. Cogn. Process..

[133] Akaike, H., Parzen, E., Tanabe, K. & Kitagawa, G. *Selected Papers of Hirotugu Akaike*, (Springer, 1998).

