# Supplementary material for: Clathrin-nanoparticles deliver BDNF to hippocampus and enhance neurogenesis, synaptogenesis and cognition in HIV/neuroAIDS mouse model
Source: Commun Biol. 2022 Mar 17;5:236. doi: 10.1038/s42003-022-03177-3 (PMC8931075; doi:10.1038/s42003-022-03177-3)
Supplement: Supplementary file 1 — Supplementary Information [file 42003_2022_3177_MOESM1_ESM.pdf]

Supplementary Information

Clathrin-nanoparticles deliver BDNF to hippocampus and enhance neurogenesis, synaptogenesis and cognition in HIV/neuroAIDS mouse model

Gordana D. Vitaliano, Jae K. Kim, Marc J. Kaufman, Christopher W. Adam, Gonzalo Zeballos, Abinaya Shanmugavadivu, Sivan Subburaju, Jay P. McLaughlin, Scott E. Lukas and Franco Vitaliano

Correspondence to: gvitaliano@mclean.harvard.edu

This PDF file includes:

Supplementary Figure 1 to 7
Supplementary Table 1 to 4

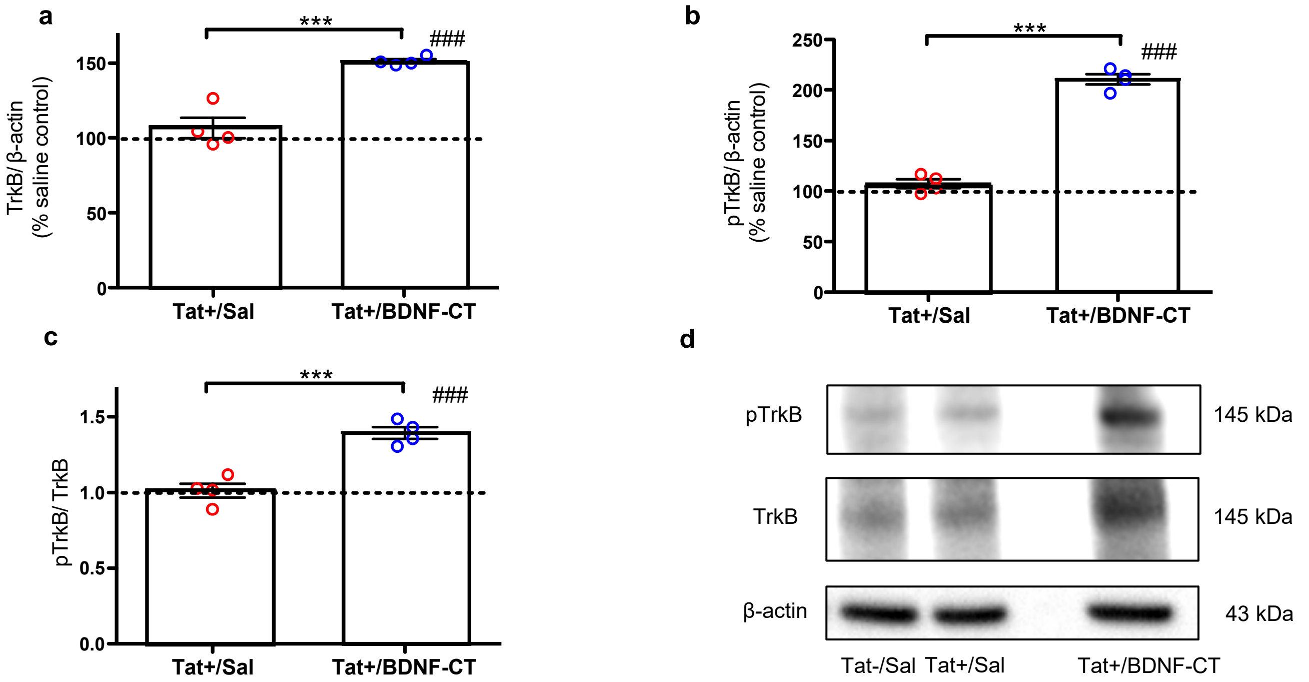

Supplementary Figure 1. BDNF-CT significantly increased TrkB expression and signaling. Tat+ mice received Dox (100 mg/kg/d, i.p.) with either i.n. Sal (Tat+/Sal) or BDNF-CT (Tat+/BDNF-CT, 0.3 mg/kg of BDNF & 2.4 mg/kg of CT) for 4 days. Tat- controls received i.p. and i.n. saline (Tat-/Sal). Western blot analysis of hippocampal tissues showed the increased expression of the full-length TrkB (TrkB) **(a)** and pTrkB **(b)** and the increased pTrkB/Trk ratio **(c)** in BDNF-CT vs. Sal treated Tat+ mice (***$p<0.001$), as well as Tat- mice (###$p<0.001$). Representative images of WB bands **(d)** are shown. Data are shown as the mean % change from Tat-/Sal controls represented by a dotted line (n=4 per group). Error bars represent S.E.M.

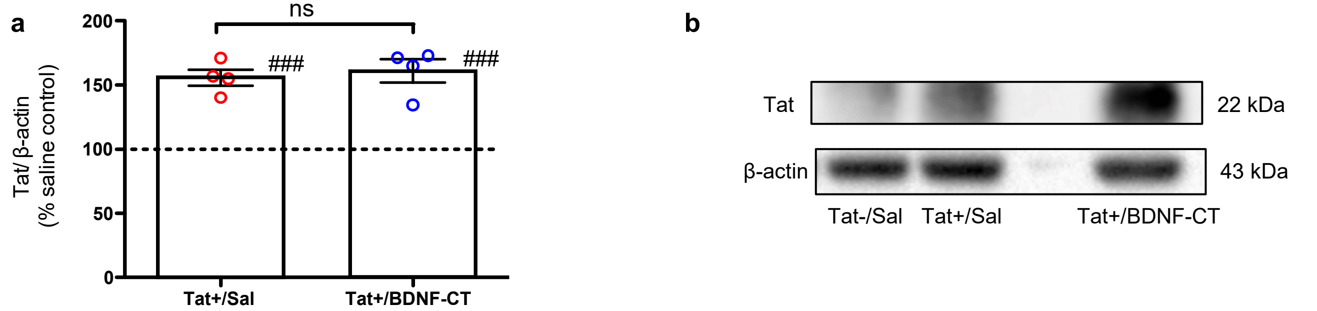

Supplementary Figure 2. Tat expression in the hippocampus of Dox-treated iTat mice was not significantly altered by BDNF-CT. Tat+ mice received Dox (100 mg/kg/d, i.p.) with either i.n. Sal (Tat+/Sal) or BDNF-CT (Tat+/BDNF-CT) for 4 days. Tat- controls received i.p. and i.n. saline. **(a)** Western blot analysis of hippocampal tissues showed the upregulaton of Tat in Tat+ (Tat+/Sal and Tat+/BDNF-CT) mice vs. Tat-/Sal mice. BDNF-CT treatment had no effect on Tat protein levels in Tat+ mice. Data are shown as the mean % change from Tat-/Sal controls represented by a dotted line (n=4 per group, ###$p<0.001$). Error bars represent S.E.M. **(b)** Representative WB images of Tat 22 kDa bands used for this analysis are shown.

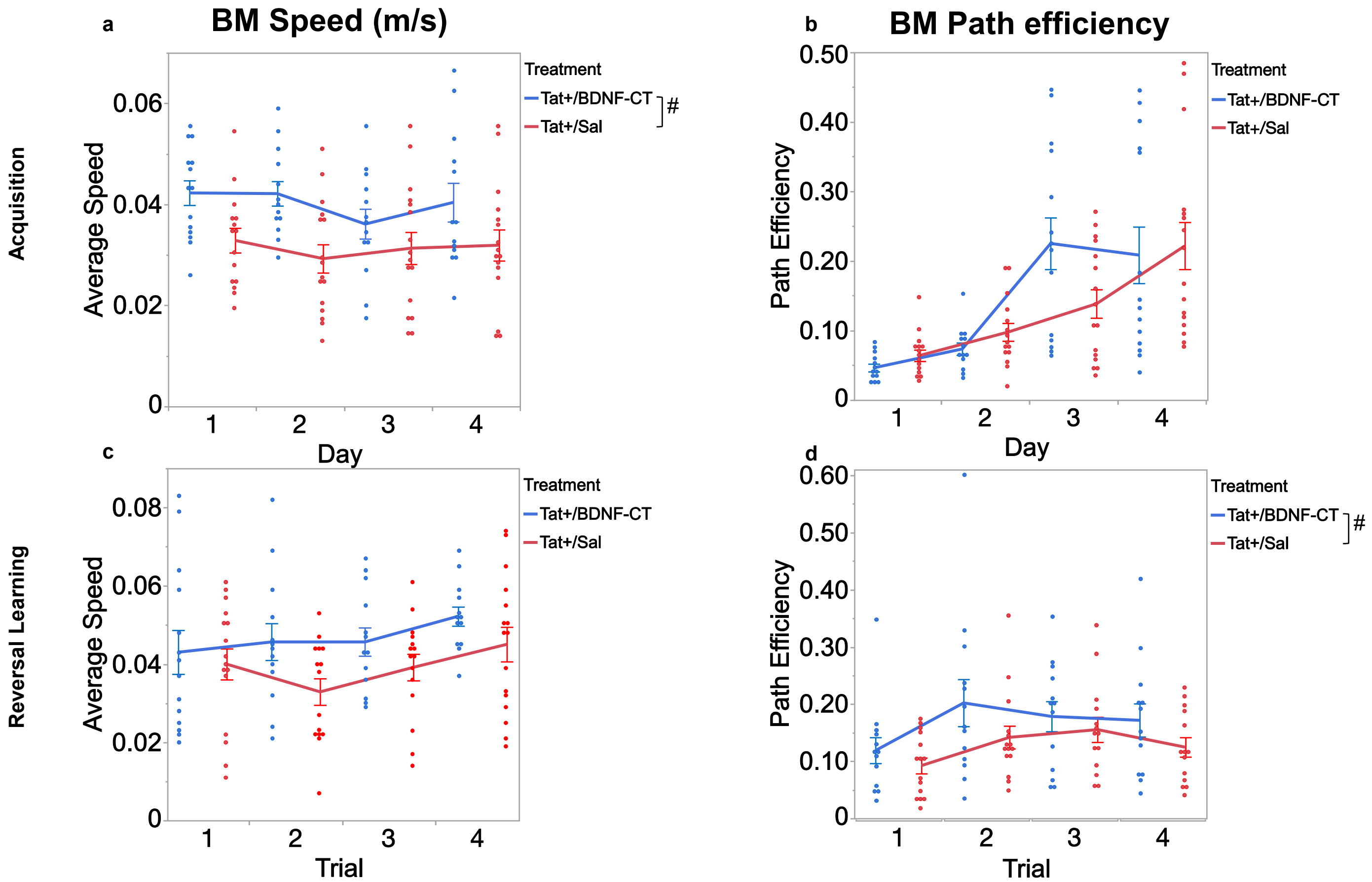

Supplementary Figure 3. BDNF-CT effects on mouse speed and path efficiency in the BMT. Average speed and path efficiency during the acquisition **(a,b)** and reversal learning phases **(c,d)** of the Barnes maze tests are shown. In the acquisition phase, average speed was higher in Tat+ mice that received BDNF-CT vs. saline (Sal) **(a)**. BDNF-CT treatment did not have a significant effect on path efficiency **(b)**. In the reversal learning phase, no overall BDNF-CT effect on average speed was found **(c).** BDNF-CT-treated Tat+ mice had higher path efficiency (**d)** compared to saline treated Tat+ mice. Significant treatment effects are indicated by $^{\#}p<0.05$. Error bars are S.E.M.

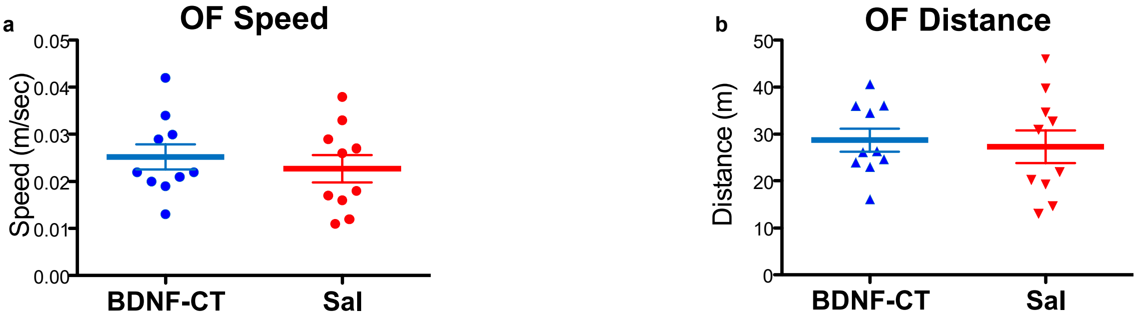

Supplementary Figure 4. BDNF-CT did not impair motor function. C57BL/6J mice received i.n. saline (n=10) or BDNF-CT (n=10) daily for 7 days. Open Field (OF) testing was performed on the 7th day and lasted 20 min. The average speed **(a)** and distance traveled **(b)** were not significantly different in BDNF-CT vs. saline treated healthy C57BL/6J mice. Error bars represent S.E.M.

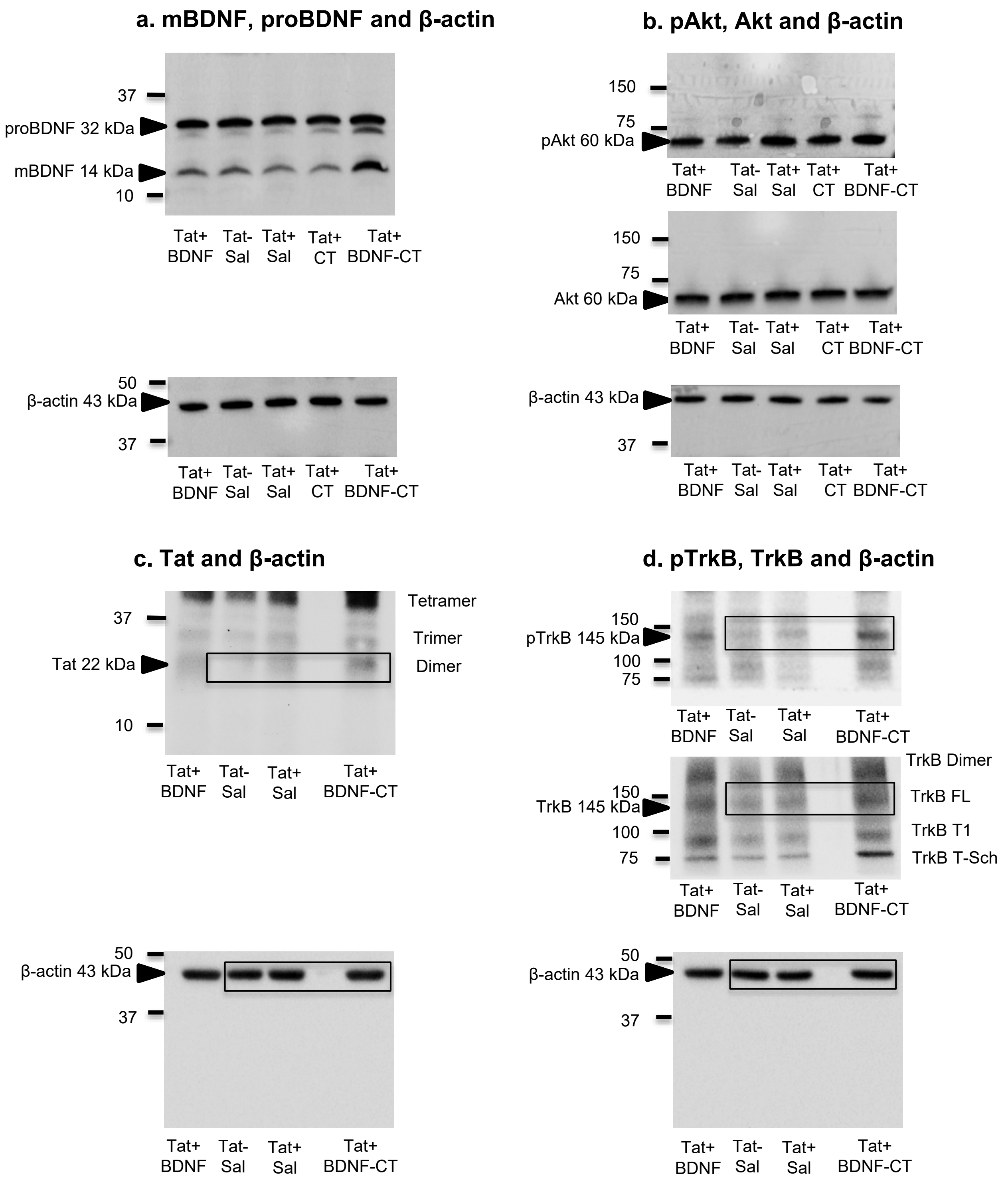

Supplementary Figure 5. Original blots for the cropped images shown in the Fig. 3 and Supplementary Figures 1 & 2. (a) mBDNF, proBDNF and β-actin; **(b)** pAkt, Akt and β-actin; **(c)** Tat and β-actin and **(d)** pTrkB, TrkB and β-actin**.** Membranes were cut at 50 kDa markings before processing.

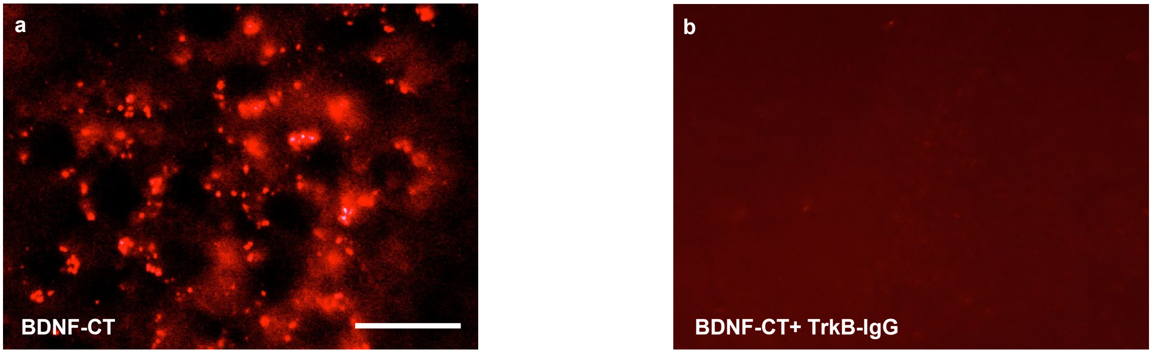

Supplementary Figure 6. BDNF-CT specificity. Rhodamine labeled clathrin nanoparticles with intact BDNF **(a)** but not with BDNF blocked with TrkB-IgG chimera **(b)** bind *in vitro* to the mouse TrkB in hippocampal slices. Scale bar is 50 μm.

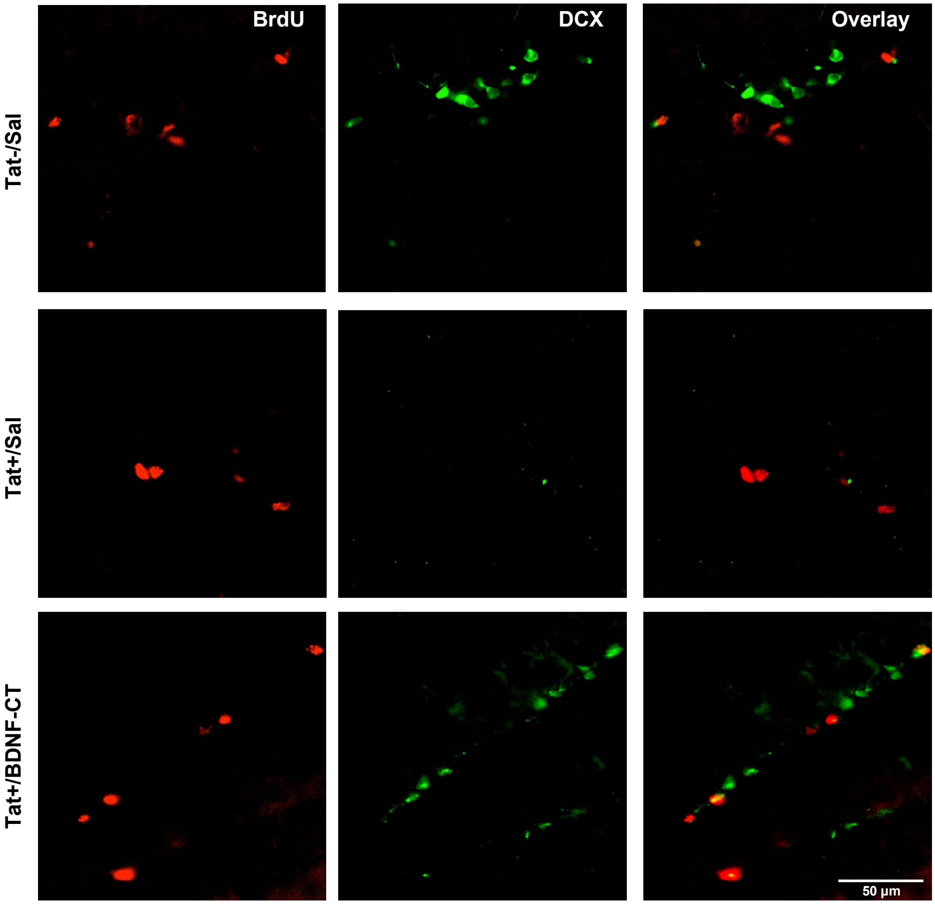

Supplementary Figure 7. Additional fluorescent mouse GCL images from different groups shown in the manuscript (Fig. 5).

Animal Experiments	Corresponding Figures	Intraperitoneal Saline or Doxycycline (Dox)	Intranasal Treatments	Tat Induction Period
Western blot analysis of mBDNF, proBDNF, pAkt, Akt, pTrkB, TrkB and Tat	Fig. 3;<br>Supp. Figs.<br>1, 2 & 5	Tat-/Sal	Sal	4 Days
		Tat+/Dox<br>(100 mg/kg/d)	Sal<br>CT<br>BDNF<br>BDNF-CT	
BDNF-CT targeting of Hippocampal TrkB	Fig. 2<br>Supp. Fig. 6	Tat+/Dox<br>(100 mg/kg/d)	BDNF-CT-Rho<br>or Sal or<br>^{3}H-CT-BDNF on day 8	7 Days
Analysis of BrdU+, Ki67+, DCX+ cell densities using IHC<br>(BrdU was given during the first 2 days of Tat-induction)	Figs. 4 & 5;<br>Supp. Fig. 7	Tat-/Sal	Sal	
		Tat+/Dox<br>(100 mg/kg/d)	Sal<br>BDNF-CT	
Analysis of SYP+ and MAP2+ IRs using IF<br>Barnes maze and novel object recognition behavior testing	Fig. 6,7 & 8;<br>Supp. Fig. 3	Tat+/Dox<br>(100 mg/kg/d)	Sal	
			BDNF-CT	

Supplementary Table 1. Summary of Tat induction paradigms and treatment groups. Unlike BDNF-CT, unconjugated BDNF did not affect mBDNF, proBDNF, pAkt, or Akt levels or the mBDNF/proBDNF or pAkt/Akt ratios, suggesting that CT are required to deliver effective BDNF concentrations. Therefore, based on *a priori* go/no-go criteria, BDNF alone groups were not used in subsequent molecular or behavioral experiments.

	Target (anti-)	Dilution	Vendor	Clone	Lot number	RRID	Product ID
Primary (IF)	TrkB	1:200	Santa Cruz Biotechnology	pAb (H181)	E0516	AB_2155274	SC-8316
	BrdU	1:100	BD Biosciences	mAb (B44)	7324574	AB_2313824	347580
	DCX	1:250	Santa Cruz Biotechnology	pAb (C18)	A0714	AB_2088494	SC-8066-R
	SYP	1:500	Santa Cruz Biotechnology	mAb (D4)	L2016	AB_628311	SC-17750
	MAP2	1:500	Cell Signaling Technology	pAb	4	AB_10693782	4542
Primary (IHC)	BrdU	1:100	BD Biosciences	mAb (B44)	7324574	AB_2313824	347580
	Ki67	1:200	BD Biosciences	mAb (B56)	6064956	AB_393778	550609
	DCX	1:100	Santa Cruz Biotechnology	pAb (C18)	I0616	AB_2088494	SC-8066
Primary (WB)	BDNF	1:200	Santa Cruz Biotechnology	pAb (N20)	K1215/G1315	AB_63094	SC-546
	Akt	1:1000	Cell Signaling Technology	mAb (C67E7)	17	AB_915783	4691
	p-Akt	1:1000	Cell Signaling Technology	mAb (D9E)	19	AB_2315049	4060
	TrkB	1:1000	Santa Cruz Biotechnology	pAb (H181)	E0516	AB_2155274	SC-8316
	pTrkB	1:1000	Cell Signaling Technology	pAb (4914)	12	AB_2298805	9141
	β-actin	1:1000	Santa Cruz Biotechnology	mAb (C4)	L0117/E0615	AB_2714189	SC-47778
	Tat	1:500	NIH AIDS Reagent Program	mAb (NT3, 2D1.1)	150254	AB_1562735	4138
Secondary (IF)	Goat-anti-rabbit Alexa Fluor 488	1:200	Fisher Scientific	pAb	1793903	AB_143165	A-11008
	Goat-anti-mouse Alexa Fluor 564	1:200	Fisher Scientific	pAb	1797971	AB_2534072	A-11004
Secondary (IHC)	Goat-anti-mouse Biotinylated	1:200	Jackson Immuno Research Lab	pAb	124967	AB_2338557	115-065-003
	Donkey-anti-goat Biotinylated	1:500	Jackson Immuno Research Lab	pAb	127296	AB_2340396	705-065-003
Secondary (WB)	Anti-rabbit IgG, HRP-linked	1:5K-10K	Cell Signaling Technology	pAb	26	AB_2099233	7074S
	Anti-mouse IgG, HRP-linked	1:5K-10K	Cell Signaling Technology	pAb	31	AB_330924	7076S

Supplementary Table 2. List of primary and secondary antibodies used. Abbreviations are as follows: Immunofluorescence (IF), immunohistochemistry (IHC), Western blot (WB), monoclonal antibody (mAb), polyclonal antibody (pAb).

Supplementary Table 3.		Animals n	Descriptive Stats (average, variance)	P Value	Degrees of freedom & F/t/z/R/ECT Value		Test Description
Test used	Figure #	Exact Value	Reported	Exact Value	Exact Value	Exact Value	
One-Way ANOVA Tukey-Kramer post hoc	3b	5, 5, 5, 4, 4	Mean +/- SEM	P= 0.0007	$F_{(4,18)}$ = 8.069	η2 = 0.642	mBDNF WB
One-Way ANOVA Tukey-Kramer post hoc	3c	5, 5, 5, 4, 4	Mean +/- SEM	p = 0.0025	$F_{(4,18)}$ = 6.239	η2 = 0.581	proBDNF WB
One-Way ANOVA Tukey-Kramer post hoc	3d	5, 5, 5, 4, 4	Mean +/- SEM	P= 0.0019	$F_{(4,18)}$ = 6.554	η2 = 0.593	mBDNF/proBDNF Ratio WB
One-Way ANOVA Tukey-Kramer post hoc	3e	5, 5, 5, 4, 4	Mean +/- SEM	P= 0.0004	$F_{(4,18)}$ = 8.874	η2 = 0.664	Akt WB
One-Way ANOVA Tukey-Kramer post hoc	3f	5, 5, 5, 4, 4	Mean +/- SEM	P< 0.0001	$F_{(4, 18)}$ = 11.720	η2 = 0.723	pAkt WB
One-Way ANOVA Tukey-Kramer post hoc	3g	5, 5, 5, 4, 4	Mean +/- SEM	P= 0.0003	$F_{(4,18)}$ = 9.314	η2 = 0.674	pAkt/Akt Ratio WB
One-Way ANOVA Tukey-Kramer post hoc	4a	4, 4, 6	Mean +/- SEM	P= 0.0002	$F_{(2,11)}$ = 21.43	η2 = 0.796	BrdU IHC
One-Way ANOVA Tukey-Kramer post hoc	4b	4, 4, 5	Mean +/- SEM	P < 0.0001	$F_{(2,10)}$ = 38.54	η2 = 0.885	Ki67 IHC
One-Way ANOVA Tukey-Kramer post hoc	4c	4, 4, 4	Mean +/- SEM	P=0.0002	$F_{(2,9)}$ = 24.69	η2 = 0.846	DCX IHC
One-Way ANOVA Tukey-Kramer post hoc	5b	3, 3, 3	Mean +/- SEM	P= 0.0003	$F_{(2,6)}$ = 43.66	ŋ2 = 0.936	DCX and BrdU IF
Unpaired t-Test (one tailed)	6d	6,8	Mean +/- SEM	P=0.0007	$t_{(12)}$=4.101	Cohen's d=0.584	SYP_DG IF
Unpaired t-Test (one tailed)	6d	6,7	Mean +/- SEM	P=0.0177	$t_{(11)}$=2.396	Cohen's d=0.343	SYP_CA1 IF
Unpaired t-Test (one tailed)	6d	6,8	Mean +/- SEM	P=0.0003	$t_{(12)}$=4.575	Cohen's d=0.636	SYP_CA3 IF
Unpaired t-Test (one tailed)	6e	6,6	Mean +/- SEM	P=0.0052	$t_{(10)}$=3.151	Cohen's d=0.498	MAP2_DG IF
Unpaired t-Test (one tailed)	6e	6,7	Mean +/- SEM	P=0.0195	$t_{(11)}$=2.341	Cohen's d=0.333	MAP2_CA1 IF
Unpaired t-Test (one tailed)	6e	6,6	Mean +/- SEM	P=0.0396	$t_{(10)}$=1.954	Cohen's d=0.276	MAP2_CA3 IF
One-Way ANOVA Tukey-Kramer post hoc	7a	10, 5, 5	Mean +/- SEM	P = 0.0343	$F_{(2,17)}$ = 4.138	η2 = 0.327	NORT
Linear Mixed Model Repeated Measure Analysis (AIC=1128.28)	7c	14, 16	Mean +/- SEM	P = 0.0467	$F_{(1,27.7)}$ = 4.336	Cohen's d = 0.804	BMT Acquisition Treatment Effect (latency)
Linear Mixed Model Repeated Measure Analysis (AIC=1210.34)	7d	14, 16	Mean +/- SEM	P = 0.0021	$F_{(1,30.9)}$ = 11.285	Cohen's d = 1.204	BMT Rev. Learning Treatment Effect (latency)
Linear Mixed Model Repeated Measure Analysis (AIC=809.56)	7g	14, 16	Mean +/- SEM	P = 0.605	$F_{(1,27.8)}$ = 0.273	Cohen's d = 0.198	BMT Acquisition Treatment Effect (# of Ref. Err.)
Linear Mixed Model Repeated Measure Analysis (AIC=673.52)	7h	14, 16	Mean +/- SEM	P = 0.0258	$F_{(1,107)}$ = 5.112	Cohen's d = 0.881	BMT Rev. Learning Treatment Effect (# of Ref. Err.)
One-Way ANOVA Tukey-Kramer post hoc	S1a	4,4,4	Mean +/- SEM	P< 0.0001	$F_{(2,9)}$ = 47.98	η2 = 0.914	TrkB WB
One-Way ANOVA Tukey-Kramer post hoc	S1b	4,4,4	Mean +/- SEM	P < 0.0001	$F_{(2,9)}$ = 255.3	η2 = 0.983	pTrkB WB
One-Way ANOVA Tukey-Kramer post hoc	S1c	4,4,4	Mean +/- SEM	P< 0.0001	$F_{(2,9)}$ = 39.78	η2 = 0.898	pTrkB/TrkB Ratio WB
One-Way ANOVA Tukey-Kramer post hoc	S2a	4,4,4	Mean +/- SEM	P = 0.0001	$F_{(2,9)}$ = 28.20	η2 = 0.862	Tat WB
Linear Mixed Model Repeated Measure Analysis (AIC=-734.19)	S3a	14, 16	Mean +/- SEM	P = 0.0120	$F_{(1,27.2)}$ = 7.243	Cohen's d = 0.984	BMT Acquisition Treatment Effect (Ave. Speed)
Linear Mixed Model Repeated Measure Analysis (AIC=-202.98)	S3b	14, 16	Mean +/- SEM	P = 0.6913	$F_{(1,28.9)}$ = 0.161	Cohen's d = 0.152	BMT Acquisition Treatment Effect (Path Efficiency)
Linear Mixed Model Repeated Measure Analysis (AIC=-643.93)	S3c	14, 16	Mean +/- SEM	P = 0.0871	$F_{(1,27.4)}$ = 3.15	Cohen's d = 0.665	BMT Rev. Learning Treatment Effect (Ave. Speed)
Linear Mixed Model Repeated Measure Analysis (AIC=-200.74)	S3d	14, 16	Mean +/- SEM	P = 0.0325	$F_{(1,43.7)}$ = 4.881	Cohen's d = 0.955	BMT Rev. Learning Treatment Effect (Path Efficiency)
Unpaired t-Test (two tailed)	S4a	10, 10	Mean +/- SEM	P=0.5355	$t_{(18)}$=0.632	Cohen's d=0.022	OF-Speed Toxic Effecs
Unpaired t-Test (two tailed)	S4b	10, 10	Mean +/- SEM	P=0.7466	$t_{(18)}$=0.3282	Cohen's d=0.006	OF-Distance Toxic Effecs

Supplementary Table 3. Satatistics Summary for Figures 3 to 7 and Supplementary Figures 1 to 4.

Supplementary Table 4.		Animals n	Correlation Coefficient r	P Value	Confidence Interval Lower 95%	Confidence Interval Upper 95%	Test Description
Test used	Figure #	Exact Value	Exact Value	Exact Value	Exact Value	Exact Value	
Pearson Product Moment Correlation	8a	12	0.688306726	0.013332418	0.189105713	0.904795654	SYP_CA1 & Reversal Latency % Change
Pearson Product Moment Correlation	8b	13	0.558642542	0.047203721	0.01106224	0.848466581	SYP_DG & Reversal Latency % Change
Pearson Product Moment Correlation	8c	13	0.581210166	0.037227742	0.044463908	0.857569049	SYP_CA3 & Reversal Latency % Change
Pearson Product Moment Correlation	8d	12	0.402463285	0.194613385	-0.222929271	0.793164307	MAP2_CA1 & Reversal Latency % Change
Pearson Product Moment Correlation	8e	11	0.716561366	0.013100165	0.204658058	0.920683221	MAP2_DG & Reversal Latency % Change
Pearson Product Moment Correlation	8f	11	0.238419136	0.480184824	-0.42177949	0.733401791	MAP2_CA3 & Reversal Latency % Change
Pearson Product Moment Correlation	8g	12	-0.569893207	0.053051599	-0.861899654	0.005956588	SYP_CA1 & Least Square Mean Errors
Pearson Product Moment Correlation	8h	13	-0.736931894	0.00405653	-0.915989901	-0.313061767	SYP_DG & Least Square Mean Errors
Pearson Product Moment Correlation	8i	13	-0.776882204	0.001782903	-0.929846208	-0.394959645	SYP_CA3 & Least Square Mean Errors
Pearson Product Moment Correlation	8j	12	-0.653075504	0.02129951	-0.892476106	-0.126637916	MAP2_CA1 & Least Square Mean Errors
Pearson Product Moment Correlation	8k	11	-0.66003763	0.027098893	-0.902554959	-0.099597101	MAP2_DG & Least Square Mean Errors
Pearson Product Moment Correlation	8l	11	-0.456267439	0.15836818	-0.829190236	0.197725807	MAP2_CA3 & Least Square Mean Errors

Supplementary Table 4. Satatistics Summary for Figure 8.
