# Supplementary material for: Clathrin-nanoparticles deliver BDNF to hippocampus and enhance neurogenesis, synaptogenesis and cognition in HIV/neuroAIDS mouse model
Source: Commun Biol. 2022 Mar 17;5:236. doi: 10.1038/s42003-022-03177-3 (PMC8931075; doi:10.1038/s42003-022-03177-3)
Supplement: Supplementary file 2 — Description of Additional Supplementary Files [file 42003_2022_3177_MOESM2_ESM.pdf]

Description of Additional Supplementary Files

Clathrin-nanoparticles deliver BDNF to hippocampus and enhance neurogenesis, synaptogenesis and cognition in HIV/neuroAIDS mouse model

Gordana D. Vitaliano, Jae K. Kim, Marc J. Kaufman, Christopher W. Adam, Gonzalo Zeballos, Abinaya Shanmugavadivu, Sivan Subburaju, Jay P. McLaughlin, Scott E. Lukas and Franco Vitaliano

Correspondence to: gvitaliano@mclean.harvard.edu

File name: Supplementary Data 1

Description: The source data for Figures 1 to 8 and Supplemental Figures 1 to 4
