# Supplementary material for: Clathrin-nanoparticles deliver BDNF to hippocampus and enhance neurogenesis, synaptogenesis and cognition in HIV/neuroAIDS mouse model
Source: Commun Biol. 2022 Mar 17;5:236. doi: 10.1038/s42003-022-03177-3 (PMC8931075; doi:10.1038/s42003-022-03177-3)
Supplement: Supplementary file 4 — Reporting Summary [file 42003_2022_3177_MOESM4_ESM.pdf]

nature research

Corresponding author(s): Gordana Vitaliano

Last updated by author(s): 01/4/2022

Reporting Summary

Nature Research wishes to improve the reproducibility of the work that we publish. This form provides structure for consistency and transparency in reporting. For further information on Nature Research policies, see our Editorial Policies and the Editorial Policy Checklist.

Statistics

For all statistical analyses, confirm that the following items are present in the figure legend, table legend, main text, or Methods section.

n/a	Confirmed	
☐	☒	The exact sample size (*n*) for each experimental group/condition, given as a discrete number and unit of measurement
☐	☒	A statement on whether measurements were taken from distinct samples or whether the same sample was measured repeatedly
☐	☒	The statistical test(s) used AND whether they are one- or two-sided<br>*Only common tests should be described solely by name; describe more complex techniques in the Methods section.*
☐	☒	A description of all covariates tested
☐	☒	A description of any assumptions or corrections, such as tests of normality and adjustment for multiple comparisons
☐	☒	A full description of the statistical parameters including central tendency (e.g. means) or other basic estimates (e.g. regression coefficient) AND variation (e.g. standard deviation) or associated estimates of uncertainty (e.g. confidence intervals)
☐	☒	For null hypothesis testing, the test statistic (e.g. *F*, *t*, *r*) with confidence intervals, effect sizes, degrees of freedom and *P* value noted<br>*Give P values as exact values whenever suitable.*
☒	☐	For Bayesian analysis, information on the choice of priors and Markov chain Monte Carlo settings
☒	☐	For hierarchical and complex designs, identification of the appropriate level for tests and full reporting of outcomes
☐	☒	Estimates of effect sizes (e.g. Cohen's *d*, Pearson's *r*), indicating how they were calculated

Our web collection on statistics for biologists contains articles on many of the points above.

Software and code

Policy information about availability of computer code

Data collection	The following data collection software packages were used in this study: Gen5 software (BioTek Instruments, Winooski, VT); PrimeView 5.31 (GE Healthcare Life Sciences, Marlborough, MA); LB-550 software (Horiba, Kyoto, Japan); Beckman LC6500 software (Beckman Coulter Life Sciences, Indianapolis, IN); Image Lab software 6 (Bio-Rad Laboratories, Hercules CA); Stereo Investigator 11 (MBF Bioscience, Williston, VT); Micro-Manager software 1.4 (https://micro-manager.org/); Fiji 1.52h (NIH, https://imagej.net/Fiji); Leica Application Suite X (Leica Microsystems, Buffalo Grove, IL), Microsoft Office 2016 for Mac and ANY-maze software 6 (Stoelting Co., Wood Dale, IL).
Data analysis	The Prism 7 (GraphPad Software Inc., La Jolla, CA) and JMP Pro 14 software package (SAS Institute Inc., Carey, NC) were used for data analyses.

For manuscripts utilizing custom algorithms or software that are central to the research but not yet described in published literature, software must be made available to editors and reviewers. We strongly encourage code deposition in a community repository (e.g. GitHub). See the Nature Research guidelines for submitting code & software for further information.

Data

Policy information about availability of data

All manuscripts must include a data availability statement. This statement should provide the following information, where applicable:

- Accession codes, unique identifiers, or web links for publicly available datasets
- A list of figures that have associated raw data
- A description of any restrictions on data availability

The source data can be found in the Supplementary Data 1. Other data that support the findings of this study are available from ExQor Technology Inc. These data are available from the authors upon reasonable request and with ExQor Technologies permission.

Field-specific reporting

Please select the one below that is the best fit for your research. If you are not sure, read the appropriate sections before making your selection.

☒ Life sciences ☐ Behavioural & social sciences ☐ Ecological, evolutionary & environmental sciences

For a reference copy of the document with all sections, see nature.com/documents/nr-reporting-summary-flat.pdf

Life sciences study design

All studies must disclose on these points even when the disclosure is negative.

Sample size	To determine the sample size in our experiments using power analysis, we followed the standard sample sizes used in similar experiments in each of the relevant fields in the literature. Supplementary Table 3 provides the exact subject number for each group in each experiment.
Data exclusions	Animals were excluded from studies for any one of the following reasons: death, major trauma, anatomical brain and/or other organ malformations, or serious Tat-induced medical problems (e.g., seizures, difficulties breathing, severe gastrointestinal problems, non-healing wounds etc.). Data values that were two standard deviations or more away from group means were a priori defined as outliers and excluded from analyses.
Replication	All attempts at replication were successful. In addition, similar results were obtained from other neurodegenerative animal models not shown in the present paper.
Randomization	For BDNF-CT WB experiments (Fig. 3), iTat mice were randomized into Tat- and Tat+ groups and were administered daily i.p. saline (Tat-controls) or Dox (100 mg/kg/d; Tat+) for 4 days. Tat+ mice were randomized into four treatment groups: saline (Tat+ /Sal), BDNF (0.3 mg/kg; Tat+/BDNF), CT (2.4 mg/kg; Tat+ /CT), or BDNF-CT (Tat+ /BDNF-CT). All i.n. treatments were administered daily 5 h after Dox i.p. injections (Fig. 3). Non-induced iTat mice (Tat-) received i.n. saline 5 h after i.p. saline injections (Sal) and were included as a global control group.<br>For neurogenesis experiments (Figs. 4 & 5), iTat mice were randomized into Tat- and Tat+ groups and were administered i.p. saline (Tat-controls) or Dox (100 mg/kg/d; Tat+) daily for 7 days. Tat+ mice were randomized into two treatment groups and were given either i.n. BDNF (0.3 mg/kg) conjugated to CT (2.4 mg/kg) (Tat+/BDNF-CT), or saline (Tat+/Sal) 5 h after their Dox injections. Tat- mice also received i.n. saline 5 h after i.p. saline injections (Sal). During the first 2 treatment days, all animals also received bromodeoxyuridine (BrdU, 50 mg/kg, i.p.) at 7 am and 7 pm (Fig. 4 & 5).<br>For synaptogenesis and behavioral studies (Figs. 6, 7 & 8), iTat mice were treated for 7 days with Dox (100 mg/kg/d, i.p.), randomized into two treatment groups and were given either i.n. BDNF conjugated to CT (Tat+/BDNF-CT), or saline (Tat+/Sal) 5 h after their Dox injections (Fig. 6).<br>Animals were randomly assigned to groups by trained researchers.
Blinding	All investigators were blinded to animal group allocation during data collection and analyses.

Reporting for specific materials, systems and methods

We require information from authors about some types of materials, experimental systems and methods used in many studies. Here, indicate whether each material, system or method listed is relevant to your study. If you are not sure if a list item applies to your research, read the appropriate section before selecting a response.

Materials & experimental systems

n/a	Involved in the study	
☐	☒	Antibodies
☒	☐	Eukaryotic cell lines
☒	☐	Palaeontology and archaeology
☐	☒	Animals and other organisms
☒	☐	Human research participants
☒	☐	Clinical data
☒	☐	Dual use research of concern

Methods

n/a	Involved in the study	
☒	☐	ChIP-seq
☒	☐	Flow cytometry
☒	☐	MRI-based neuroimaging

Antibodies

Antibodies used	We generated a table (Supplementary Table 2) that lists antibodies used in the manuscript, their clones, catalog and lot numbers, conditions of utilization, and the Resource Identification Portal numbers (https://scicrunch.org/resources). The RRID Portal provides information regarding an antibody profile, validation statements and relevant citations.
Validation	Commercial primary antibodies were validated by manufacturers and this information was found in manufacturer's product sheets and RRID Portal. Additional validation of anti-HIV-1 Tat antibody (NT3, 2D1.1, NIH AIDS reagent program) was performed in i.p. saline vs. Dox (100 mg/kg/day i.p.) treated iTat mice using western blotting and presented in Supplementary Figure 2.

Animals and other organisms

Policy information about studies involving animals; ARRIVE guidelines recommended for reporting animal research

Laboratory animals	Adult iTat male 10-14 week-old mice were used in these studies.
Wild animals	This study did not involve wild animals.
Field-collected samples	This study did not involve samples collected from field research.
Ethics oversight	Studies were conducted using procedures approved by McLean Institutional Animal Care and Use Committee (protocols #2016N000595 and 2017N000127). All procedures conformed to NIH and National Research Council guidelines on the care and use of laboratory animals.

Note that full information on the approval of the study protocol must also be provided in the manuscript.
